# Precise fine-turning of *GhTFL1* by base editing tools defines ideal cotton plant architecture

**DOI:** 10.1186/s13059-024-03189-8

**Published:** 2024-02-26

**Authors:** Guanying Wang, Fuqiu Wang, Zhongping Xu, Ying Wang, Can Zhang, Yi Zhou, Fengjiao Hui, Xiyan Yang, Xinhui Nie, Xianlong Zhang, Shuangxia Jin

**Affiliations:** 1 National Key Laboratory of Crop Genetic Improvement, Hubei Hongshan Laboratory, Huazhong Agricultural University, Wuhan, Hubei 430070 China; 2 Key Laboratory of Oasis Ecology Agricultural of Xinjiang Production and Construction Corps, Agricultural College, Shihezi University, Shihezi, Xinjiang 832003 China

**Keywords:** Cotton, Genome editing, Base editor, Directed evolution, Plant ideotype

## Abstract

**Background:**

CRISPR/Cas-derived base editor enables precise editing of target sites and has been widely used for basic research and crop genetic improvement. However, the editing efficiency of base editors at different targets varies greatly.

**Results:**

Here, we develop a set of highly efficient base editors in cotton plants. GhABE8e, which is fused to conventional nCas9, exhibits 99.9% editing efficiency, compared to GhABE7.10 with 64.9%, and no off-target editing is detected. We further replace nCas9 with dCpf1, which recognizes TTTV PAM sequences, to broaden the range of the target site. To explore the functional divergence of TERMINAL FLOWER 1 (TFL1), we edit the non-coding and coding regions of *GhTFL1* with 26 targets to generate a comprehensive allelic population including 300 independent lines in cotton. This allows hidden pleiotropic roles for *GhTFL1* to be revealed and allows us to rapidly achieve directed domestication of cotton and create ideotype germplasm with moderate height, shortened fruiting branches, compact plant, and early-flowering. Further, by exploring the molecular mechanism of the *GhTFL1*^L86P^ and *GhTFL1*^K53G+S78G^ mutations, we find that the *GhTFL1*^L86P^ mutation weakens the binding strength of the GhTFL1 to other proteins but does not lead to a complete loss of GhTFL1 function.

**Conclusions:**

This strategy provides an important technical platform and genetic information for the study and creation of ideal plant architecture.

**Supplementary Information:**

The online version contains supplementary material available at 10.1186/s13059-024-03189-8.

## Background

Heredity and variation are the basis of plant species evolution and mordent crop breeding. The major purpose of plant breeding is to create and utilize these genetic variations [1]. However, conventional crop breeding and modern genetic engineering cannot meet the increased demand of development of human society and rapid climate changes. At present, breakthroughs in biotechnology such as CRISPR-based gene editing and bioinformatics technology are opening the door to crop “design breeding” and the “third green revolution” in agriculture [2]. CRISPR/Cas9 genome editing is widely used in many crop species to cut target DNA sites, causing double-strand breaks (DSB) to create mutants with protein function loss [3]. However, DSB causes major damage to cells, which may cause cell apoptosis, and the loss of some genes related to growth and development may lead to some traits being defective, even producing lethal mutations, which hinders its application in crop improvement. For example, the *TFL1* gene is pleiotropic and dose-sensitive and its complete inactivation using CRISPR/Cas9 knockout mostly results in the appearance of extreme traits (see below). Therefore, it is urgent to find new strategies or approaches to achieve precise regulation of architecture-related genes such as *TFL1* and achieve the goal of ideal plant shape shaping.

The base editing is a powerful tool to create point mutations in promoters, coding sequences, and upstream open reading frames (uORFs) to fine-tune quantitative traits without inducing DSB and DNA repair templates [4, 5]. The commonly used base editors, such as the cytosine base editor (CBE) and adenine base editor (ABE), enable precise base mutations of C-to-T and A-to-G without inducing DSB [6, 7]. Since the base editing system was developed, it has been widely used in various crops, including rice [8–11], corn [12, 13], wheat [10, 12, 14, 15], cotton [16, 17], and others. At present, base editing is one of the most powerful tools to study gene function, artificially evolving functional proteins, mine new gene loci, and create new germplasm resources through high-throughput point mutation. The traditional ABE is a dimer protein formed by the fusion of nCas9 and adenine deaminase composed of a wild-type Escherichia coli tRNA adenosine deaminase (TadA) and an evolutionary TadA (TadA7.10), which is guided to the target site by gRNA and realizes the single base conversion from A to G within editing window [7]. Although ABE7.10 has been successfully applied to different plants, its editing efficiency is generally low and varied among different targets, which hinders its wide application in precise plant genome editing and crop breeding [18]. Therefore, the efficiency of ABE still needs to be improved. Recently, TadA was further evolved to ABE8e using a phage-assisted non-continuous and continuous evolution (PANCE and PACE) system [19]. Compared to ABE7.10, ABE8e contains one new TadA variant, TadA8e, which has eight additional mutations in amino acid sequence over TadA7.10. After detection, ABE8e catalyzed DNA deamination about 1100 times faster than the early ABEs and has a higher compatibility with various Cas9 or Cas12 in human cells [19, 20]. Recently, ABE8e has been tested and showed efficient A-to-G editing in rice [21, 22] and wheat [23]. However, these ABE8e tools tested in plants consist of Cas9 nickase (nCas9(D10A)) which recognizes NGG PAM sequence at the target genome region, whereas, Cas12a recognizes TTTV PAM sequences. Compared to the TadA7.10 fused with dCas12a, TadA8e fused to dCas12a enhanced editing efficiency in human cells [19].

Cotton is one of the world’s most important cash crops and an important source of natural fiber [24, 25]. The climatic conditions of cotton production and the high degree of mechanization require cotton architecture to be compact and concentrated boll opening [26, 27]. Plant architecture is a complex agronomic trait determined by multiple genes, including branching patterns (number of branches, angle of branches to the main stem, degree of internode elongation, etc.) and shoot characteristics (size, shape, and position of leaves, branches, and flowers). Among these, morphogenesis at both the nutritional and reproductive growth stages plays an important role in regulating plant architecture. Flowering is a symbol of the transition from nutritional to reproductive growth in plants and the key regulators of the controlling flowering transition and the fate of shoot apical meristems (SAMs) are the phosphatidylethanolamine-binding protein (*PEBP*) gene family members florigen *FT* and antiflorigen *TFL1* [2, 28]. So far, studies on a variety of plant architecture have shown that the *TFL1* gene has a relatively conserved role in influencing inflorescence structure by preventing the differentiation of shoot meristems into floral meristems, making it a target gene for crop architecture improvement. In this study, we develop the new cotton adenine base editor GhABE8e, which compromised codon optimization TadA8e (V106W) deaminase fused with the Cas9n or dCpf1 (dead LbCas12a). We also demonstrated that pooled sgRNAs strategy with GhABE8e generated high throughput mutagenesis at target sites was useful for artificially evolving functional proteins in plants. Using these new base editors, we explored an unprecedented strategy to effectively fine-tune target gene *GhTFL1* in cotton plants by high-throughput method editing non-coding and coding regions, and architecture-remodeled cotton plants were obtained.

## Results

### Development of the high-activity GhABE8e toolbox

**Fig. 1:**
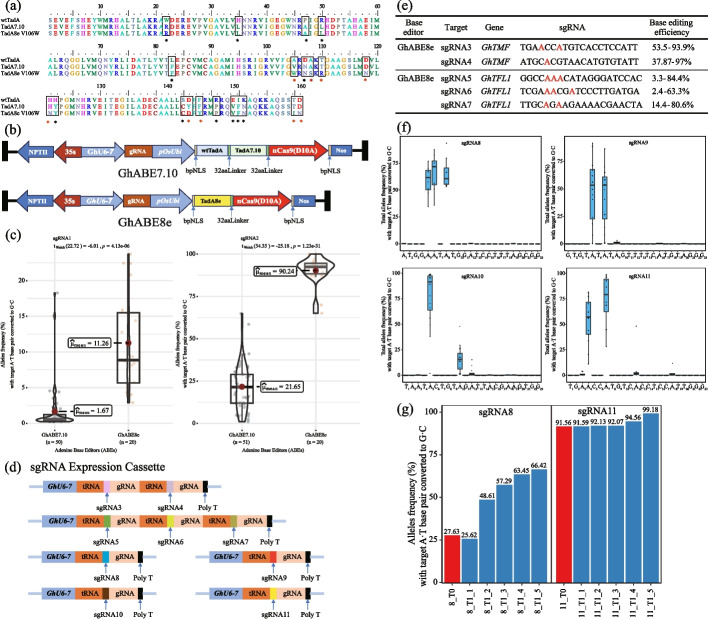
Comparison of GhABE7.10 and GhABE8e for A-to-G base editing efficiency in cotton. **a** Sequence comparison of wtTadA, TadA7.10, and TadA8e (V106W). Black boxes and black asterisks mark amino acid sites with differences between wtTadA and TadA7.10; red asterisks mark amino acid sites with differences between TadA7.10 and TadA8e (V106W). **b** Schematic diagram of the vector element of GhABE7.10 and GhABE8e. GhU6-7, Upland cotton U6 promoter; gRNA, tRNA-20bp target-gRNA; pOsUbi, rice ubiquitin 1 promoter; wtTadA, wild-type *E. coli* TadA gene; TadA7.10, TadA8e, engineered TadA genes; NLS, nuclear localization signal; NOS, nopaline synthase terminator. **c** Comparison of adenine editing efficiencies of all A-to-G conversion between GhABE7.10 and GhABE8e within sgRNA1 and sgRNA2 target region by amplicon deep sequencing. Each dot represents the editing efficiency of an independent sample. The dashed line represents the average of the editing efficiency of all samples. **d** Schematic depiction of the target element of GhABE8e for sgRNA3 to sgRNA11. **e** Base-editing efficiencies in multiple sites of GhABE8e at sgRNA3-sgRNA7. The base editing efficiency is the percentage of reads with target A•T to G•C substitution in total reads. The edited A site is indicated in red. **f** Editing windows of GhABE8e at sgRNA8-sgRNA11 in cotton. The middle line of the box represents the median and the bottom and top lines of the box represent the upper and lower quadrilles of the data, respectively. Tail extends to the minimum and maximum of data. **g** Editing efficiency of GhABE8e + sgRNA8 (left) and GhABE8e + sgRNA11 (right) in T0 and corresponding T1 seedlings

To explore a new version of ABEs with new properties in cotton, our previous ABE system, GhABE7.10 was updated by replacing the adenine deaminase wtTadA-TadA7.10 with the cotton codon-optimized TadA8e (V106W), generating a new adenine base editor, GhABE8e (Fig. 1a). To compare the editing efficiency of TadA8e and TadA7.10 in cotton, we constructed GhABE7.10 [16] and GhABE8e vectors and both of them have the nCas9 nickase. Two sgRNAs, designed for *GhPEBP*, were constructed into one vector (tRNA-sgRNA1-tRNA-sgRNA2), which were previously edited using GhABE7.10 (Fig. 1b). To investigate the efficiency and specificity of GhABE8e in cotton, a construct without sgRNAs (empty vector) was also generated as a negative control. Finally, theses constructed vectors were introduced into cotton cells by *Agrobacterium*-mediated transformation (Additional file 1: Fig. S1). The performance of these vectors in independent transgenic cotton plants was assessed using the targeted deep sequencing.

To compare the base editing efficiency of GhABE8e and GhABE7.10, the editing efficiency at sgRNA1 and sgRNA2 target sites was tested by target deep sequencing. At the sgRNA2 target site of *GhPEBP*, the efficiency of GhABE8e (60 to 99.9%, 90.2% on average) was fourfold higher than that of GhABE7.10n (5 to 64.9%, 21.7% on average [16]; Fig. 1c). Pooled sgRNAs to generate robust mutagenesis simultaneously at multiple target sites can improve the efficiency of saturation mutagenesis for high-throughput functional assessment of nucleotide variation within genes [29–31]. However, when using two sgRNAs (tRNA-sgRNA1-tRNA-sgRNA2) in ABE7.10, the editing efficiency of the 3′ end sgRNA (sgRNA2) was usually higher than that of the 5′ end sgRNA (sgRNA1) [16]. In this report, the efficiency of GhABE8e at the sgRNA1 target site, 11.3% on average, was obviously higher than that using GhABE7.10, 1.7% on average (Fig. 1c). This indicates that compared to GhABE7.10, GhABE8e is more efficient for synchronous editing of multiple sgRNA sites. To further evaluate the editing efficiency by GhABE8e at multiple loci, another five targets (tRNA-sgRNA3-tRNA-sgRNA4, tRNA-sgRNA5-tRNA-sgRNA6-tRNA-sgRNA7) were designed and tested in cotton plants. Using these sgRNA transcription cassettes (Fig. 1d and Additional file 2: Table S1), we observed high editing efficiencies at more than 90% target sites, with editing rates ranging from 33.8 to 93.9% at sgRNA3 target site; 37.87 to 97.0% at sgRNA4; 3.3 to 84.4% at sgRNA5; 2.4 to 63.3% at sgRNA6; and 14.4 to 80.6% at sgRNA7 (Fig. 1e and Additional file 1: Fig. S2). These results show that the editing activity of GhABE8e is unaffected by simultaneous targeting of multiple genomic loci.

To analyze the editing window of GhABE8e, another set of four sgRNAs (sgRNA8, sgRNA9, sgRNA10, and sgRNA11) with adenine spanning from the first nucleotide to the twelfth nucleotide in the protospacer from the 5′end were designed and tested in this report (Fig. 1d and Additional file 2: Table S1). Notably, targeted deep sequencing of edited lines shows that the editing window of GhABE8e was expanded, ranging from position A4 to A10 (counting the PAM as positions 21–23) (Fig. 1f), which is wider than the window of GhABE7.10 (Position A5). Furthermore, GhABE8e can simultaneously edit multiple adenines at a single sgRNA target site (Fig. 1f). These suggest that GhABE8e has higher deamination activity with a wider editing window.

Targeted deep sequencing of more than 200 T0 plants showed that GhABE8e can efficiently perform base editing in cotton. Then, to evaluate whether these A-to-G mutations at target sites could be inherited through the germline, the genotypes of T1 plants from GhABE8e + sgRNA8 and GhABE8e + sgRNA11 were examined by targeted deep sequencing analysis. The data showed that all T1 plants carried A-to-G or T-to-C base mutations at the sgRNA8 target site with an average of 52.28% editing efficiency and 93.91% at the sgRNA11 target site, respectively. Apparently, the editing efficiency of T1 plants carrying GhABE8e unit was higher than that of T0 parental plants, indicating that some new editing events or more cells with the same editing were generated in the T1 plants (Fig. 1g). At the same time, ten T1 generation lines were collected for positive identification and the results showed that nine of the T1 generation plants tested carried GhABE8e and one transgene-free line was identified, which showed 99.18% editing efficiency at target sgRNA11 site (Additional file 1: Fig. S3). The availability of transgene-free lines with editing confirmed that the mutation produced by GhABE8e could be inherited from the T0 parent to the progeny.

### TadA8e is compatible with dCpf1 protein for efficient base editing

In our previous ABE system, GhABE7.10-dCpf1, a fusion of dCpf1 (deactivated Cpf1) and adenine deaminase TadA7.10, was developed. The editing efficiency was low ranging from 0.2 to 0.5%, which may be due to the incompatibility between TadA7.10 and dCpf1 [16]. TadA8e showed efficient A-to-G base editing activity with dLbCas12a (dLbCpf1) for the first time in animal and plant cells [19, 32–35]. In order to further explore the compatibility of TadA8e with dCpf1 and expand the target range (PAM sites) in the cotton genome, the GhABE8e-dCpf1 vector was constructed and tested in this report (Additional file 1: Fig. S4a). One crRNA with TTTV-PAMs (crRNA12) targeting the *GhMAX1*, the member of the *CYP711A* cytochrome P450 family and a specific repressor of vegetative axillary buds generated by the axillary meristem gene, was designed for evaluating the performance of GhABE8e-dCpf1 in cotton (Additional file 2: Table S1). GhABE8e-dCpf1 exhibited an editing efficiency of 1.5% at the crRNA12 target site, which is lower than that of the GhABE8e-Cas9n system (Additional file 1: Fig. S4b and S4c). These results show that TadA8e is compatible with dCpf1 and enable to edit target genome regions with TTTV PAM sites and also reveal that TadA8e has higher compatibility with the Cas9n system than with dCpf1.

### GhABE8e achieves clean editing at the DNA and RNA level

**Fig. 2:**
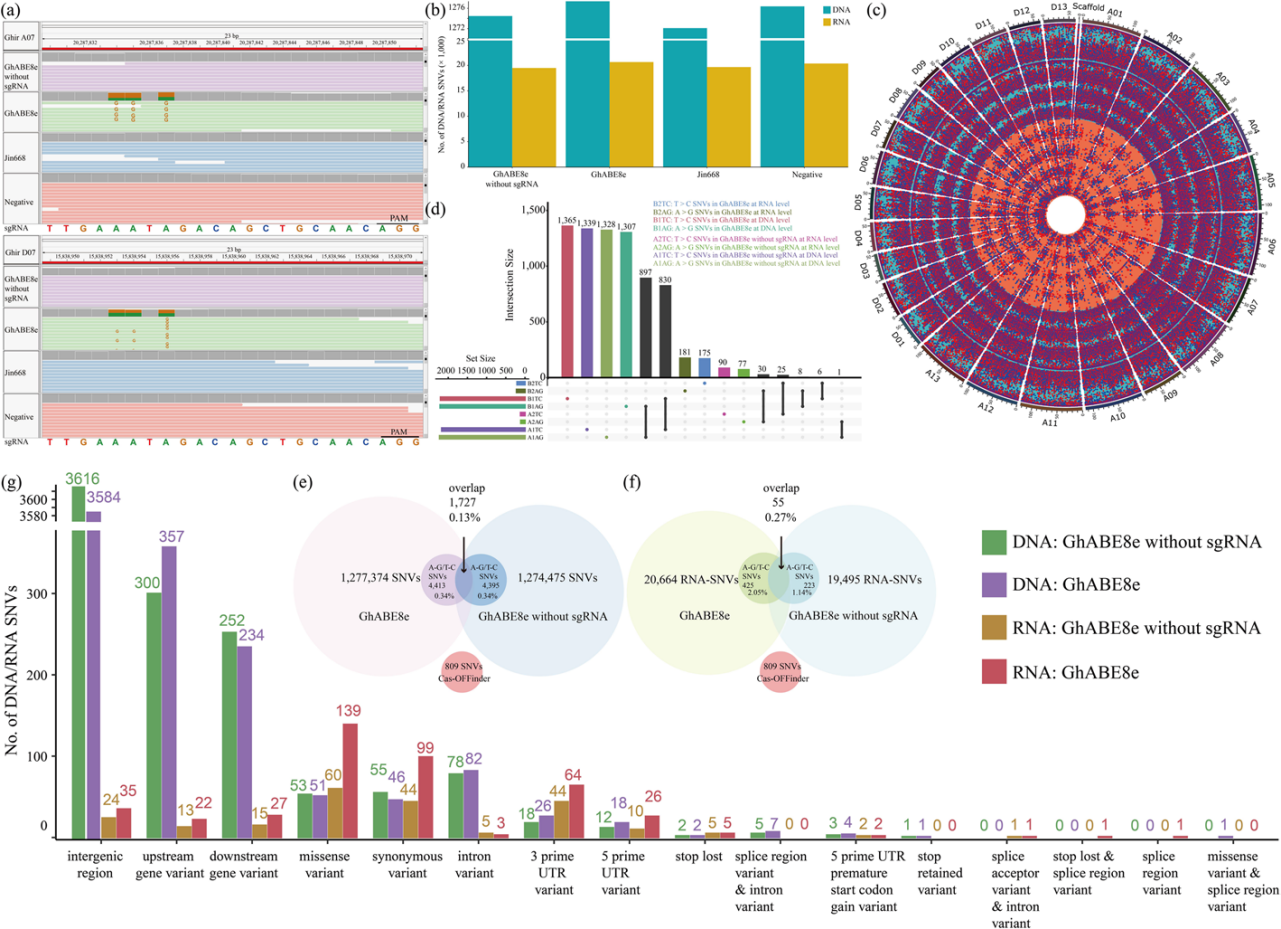
Genome-wide analysis of DNA and RNA off-target effect for the GhABE8e system by whole genome sequencing and whole transcriptome sequencing. **a** Sequence alignment of sgRNA13 on cotton genome by IGV browser views. The A-to-G mutations edited by GhABE8e were detected at the A5, A6, and A8 of target region. The target sgRNA sequences are highlighted in different colors. **b** Numbers of total SNVs identified in the GhABE8e, GhABE8e without sgRNA, WT, and negative plants. **c** Distribution characteristics of DNA and RNA off-target SNVs (A-to-G/T-to-C) on cotton chromosomes in GhABE8e without sgRNA and GhABE8e edited plants. **d** UpSet diagram of off-target SNVs (A-to-G/T-to-C) at DNA and RNA levels in GhABE8e without sgRNA and GhABE8e edited plants. **e** Venn diagrams of DNA-SNVs and Cas-OFFinder predicted off-target sites for edited lines. **f** Venn diagrams of RNA-SNVs and Cas-OFFinder predicted off-target sites for edited lines. **g** The number of different locations of SNVs for GhABE8e without sgRNA and GhABE8e-edited T0 plants at DNA and RNA levels

Drawing from prior reports [16], a comprehensive evaluation of the off-target effects of GhABE8e was conducted at both DNA and RNA levels based on the whole-genome sequencing (WGS) and whole-transcriptome sequencing (WTS). Briefly, a total of 4 plants were chosen for WGS and WTS with 50 × sequencing depth, respectively, including an edited plant generated by the GhABE8e, a positive control plant expressing GhABE8e without sgRNAs (empty vector), a negative (following tissue culture and plant generation but without T-DNA insertion) and a wild type (WT, Jin668). First, the editing specificity of GhABE8e at the DNA level was analyzed according to the WGS results. Consistent with target deep sequencing data, the A-to-G on-target mutations at the sgRNA13 target site in the plant carrying the GhABE8e system were identified by Integrative Genomics Viewer (IGV) and removed in the following off-target analysis (Fig. 2a). Through counting the number of single nucleotide variations (SNVs), 1277374, 1274475, 1276379, and 1272096 SNVs were found in the edited, positive control, negative and wild-type plants, respectively. Compared to the other three plants, plants harboring GhABE8e did not show more SNVs (Fig. 2b). Subsequently, the SNVs present in negative and wild-type individuals were used as background mutations, and those in the edited and positive control individuals were filtered out. Likewise, considering the adenine deaminase in the ABE vector mainly causes the base mutation of A-to-G and T-to-C, the subsequent analysis only focuses on the mutation of A-to-G and T-to-C [36]. For simplicity, we referred to the A-to-G/T-to-C SNVs as SNVs throughout our research. To evaluate whether the production of SNVs was dependent on sgRNA, statistical analysis was conducted on the SNV from the edited and positive control plants. The results showed that the number of SNVs in the edited and positive control plants was similar, with an edited plant of 4413 and a positive control plant of 4395, accounting for 0.34% and 0.34% of all SNVs in the corresponding plants (Fig. 2e). Meanwhile, the SNVs identified in these two plants were not found to overlap with the 809 potential off-target mutations predicted by Cas-OFFinder [37], indicating that GhABE8e did not induce sgRNA-dependent off-target editing in cotton (Fig. 2e and Additional file 2: Table S2). The SNVs identified in GhABE8e edited plants were subsequently mapped to the cotton genome, revealing a random distribution across the chromosomes with no detected mutation hotspots (Fig. 2c). Further annotation of the SNVs revealed that they were mainly distributed in intergenic regions that did not affect gene function (Fig. 2g). Therefore, these data indicated that random SNVs are unlikely to affect gene function and cause potential off-target effects in cotton.

Then, the editing specificity of GhABE8e at the RNA level was analyzed according to the WTS results. From transcriptome data, 20,664, 19,495, 20,380, and 19,675 RNA-SNVs were identified in the edited, positive control, negative, and wild-type individuals, respectively (Fig. 2b). Overall, there was no obvious difference in the number of RNA-SNVs detected among the four groups. After filtering out background mutations identified in negative and WT plants, a total of 445 and 223 RNA-SNVs were respectively identified in the edited and positive control individuals and did not concur with predicted 809 potential off-target sites by the Cas-OFFinder, indicating that GhABE8e did not induce sgRNA-dependent off-target editing at RNA level in cotton (Fig. 2f). Similar to the randomness of DNA-SNVs, the distribution of RNA-SNVs on the cotton genome did not show hotspots (Fig. 2c). By comparing the expression level between genes randomly selected from transcriptome and genes containing RNA SNVs identified in GhABE8e-edited plants, it is found that these RNA-SNVs are greatly enriched in genes with high transcription level (Additional file 1: Fig. S5).

Upon comparing the DNA-SNVs and RNA-SNVs produced by GhABE8e with and without sgRNA, a total of 15 SNVs were identified to be present in both RNA and DNA sequence (Fig. 2d). These RNA-SNVs were not caused by off-target effects of the vector, but rather, they arose from transcription at the DNA level. Comparison and analysis of DNA-SNVs and RNA-SNVs between the edited and positive control individuals revealed that 1727 DNA-SNVs overlapped, which accounted for 0.1% of total DNA-SNVs in the edited individual. Correspondingly, there were 55 RNA-SNVs overlapping in these two individuals, which accounted for 0.3% of total RNA-SNVs in the edited individual (Fig. 2f).

In summary, GhABE8e does not produce sgRNA-dependent off-target mutations at either the DNA or RNA level in cotton, and the DNA-SNVs and RNA-SNVs caused by the deaminase account for only 0.1% and 0.3% of the total SNVs.

### GhABE8e drives rapid directed evolution of *GhTFL1* to generate ideotype cotton plants

To create an ideal plant architecture in cotton, we edited the *GhTFL1* genes via CRISPR/Cas9 which resulted in the appearance of extreme traits with dwarfing and apical flowering (Additional file 1: Fig. S6). Then, the strategy based on base editor-GhABE8e was selected to achieve precise fine-tuning of *GhTFL1* genes to create a new cotton germplasm with moderate height and compact architecture.

**Fig. 3:**
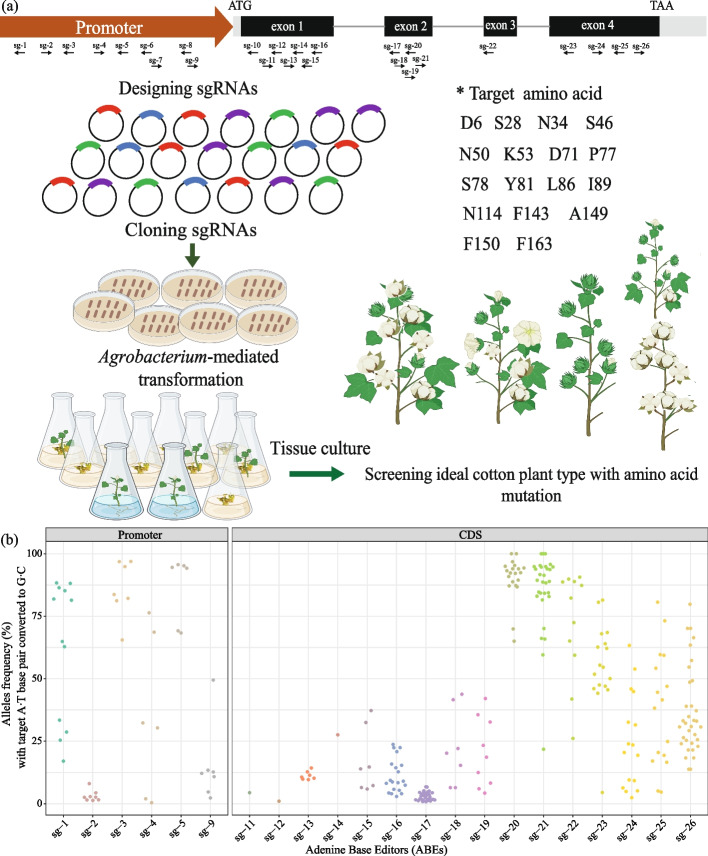
Procedure for artificially directed evolution of plant functional proteins through base editing. **a** Schematic of the procedure for artificial evolution of the CDS and promoter of *GhTFL1* via GhABE8e. Created with BioRender.com. **b** Statistics of editing efficiency for artificial evolution of the CDS and promoter of *GhTFL1* via GhABE8e

There are many web applications that design sgRNAs for CRISPR/Cas system, however, the design of sgRNAs for the base editor is more complex than that for CRISPR/Cas, which should consider editing window, amino acid changes after base mutation, off-target effect and so on. In this study, a user-friendly application BEsgRNADe was developed for the sgRNAs design of the base editor (Additional file 1: Fig. S7). According to the 525-bp open reading frame of the *GhTFL1* gene in the cotton genome, a total of 17 sgRNAs were designed using BEsgRNADe and Phyre (http://www.sbg.bio.ic.ac.uk/phyre2/html/page.cgi?id=index), which were located on the forward and reverse DNA strands, respectively (Additional file 1: Fig. S8 and Additional file 2: Table S1). In order to induce the diversity of mutated *GhTFL1* sequence by GhABE8e, we constructed vectors with dual and triple targets in the CDS region. In addition, 9 sgRNAs were designed by PlantCARE (https://bioinformatics.psb.ugent.be/webtools/plantcare/html/) targeting to the 800-bp promoter region of *GhTFL1* gene and constructed into the GhABE8e vector (Additional file 1: Fig. S9 and Additional file 2: Table S1), followed by genetic transformation in cotton. Transgenic cotton T0 plants were generated through *Agrobacterium*-mediated transformation and somatic embryogenesis (Fig. 3a and Additional file 1: Fig. S1). To explore the sequence diversification of *GhTFL1* caused by GhABE8e, genomic DNA were extracted from the callus of more than 150 independent lines. Deep sequencing of the endogenous *GhTFL1* gene in these samples showed that all callus tested harbored A-to-G/T-to-C mutations in the target region (Fig. 3b), suggesting that GhABE8e is efficient and capable of generating a large number of mutations that underlie the directed evolution of endogenous genes in the cotton genome.

**Fig. 4:**
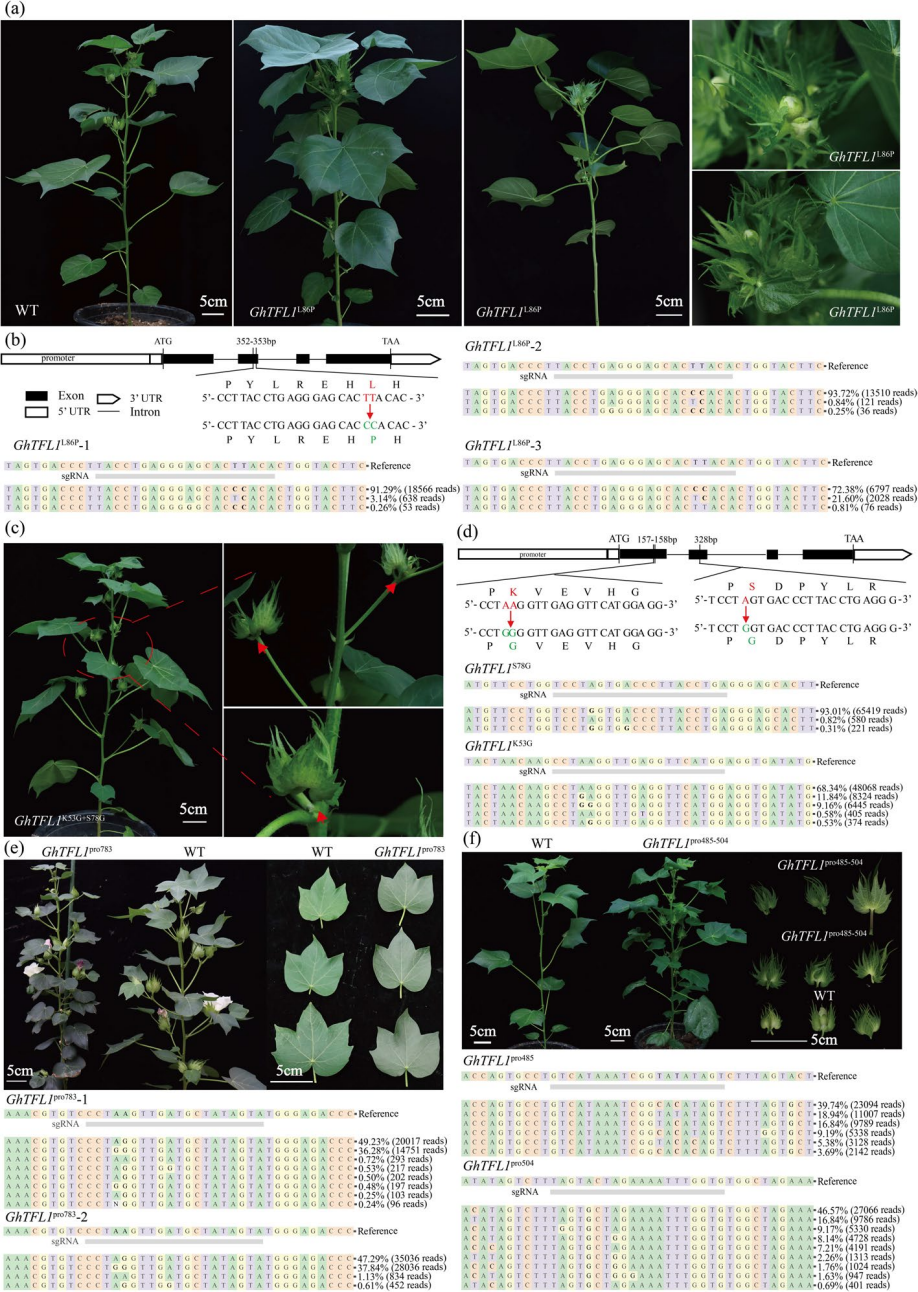
The artificial evolution of *GhTFL1* mediated by base editing generates novel plant architecture cotton mutants. **a** Mutants of the GhABE8e-induced L86P amino acid substitution in the CDS of the *GhTFL1* gene exhibit an early flowering, compact inflorescence phenotype. Scale bar, 5 cm. *GhTFL1*^L86P^ mutants develop rare twin flowers. **b** The T-to-C base editing led to amino acid substitution from leucine to proline at amino acids position 86 of *GhTFL1* and the editing efficiency is analyzed by CRISPResso2 for quantification. Three plants tested carried two types of mutations and were chimeric. **c** The T0 seedlings carrying the K53G and S78G mutations had a double ball phenotype. Scale bar, 5 cm. **d** The A-to-G base editing led to amino acid substitution from Lysine to Glutamicacid/Glycine and Serine to Glycine at amino acids position 53 and 78 of *GhTFL1* and the editing efficiency is analyzed by CRISPResso2 for quantification. This plant tested carried two types of mutations and was chimeric. **e** The plants with base editing of *GhTFL1* promoter at -783 bp showed that leaves become more numerous and smaller, with darker leaves and more nutritional growth, and the editing efficiency is analyzed by CRISPResso2 for quantification. Scale bar, 5 cm. Two plants tested carried two types of mutations and were chimeric. **f** The plants with base editing of *GhTFL1* promoter at -485-504 bp showed excessive nutritional growth and the editing efficiency is analyzed by CRISPResso2 for quantification. Scale bar, 5 cm. This plant tested carried two types of mutations and was chimeric

In total, more than 200 independent T0 generation plants were produced by *Agrobacterium*-mediated transformation using the above 26 vectors that target the *GhTFL1* gene. Editorial analysis indicates that a majority of the T0 generation plants are predominantly chimeric, with the mutation sites predominantly located within the A4 to A6 region of the window (Additional file 2: Table S3). Some plants exhibiting obvious phenotypes, such as changes in inflorescence structure, plant height, or architecture, were isolated for further analysis (Fig. 4 and Additional file 1: Fig. S10). Specifically, the plants *GhTFL1*^L86P^ carried two non-synonymous mutations (from T to C) at the 352-bp and 353-bp positions, resulting in an amino acid substitution from L to P at the 86th position. These plants showed determinate main shoots with clustered fruiting bolls and rare twin flowers. The editing efficiency of the plants’ *GhTFL1*^L86P^ mutation was up to 94.8% (Fig. 4a, b and Additional file 2: Table S4). Another plant carrying the non-synonymous mutation (from A to G) at the 157–158 bp and 328 bp position, which resulted in amino acid substitution from K to G at the 53th and S to G at the 78th position, respectively, showed a dual boll phenotype compared with wild type plants. What is more, the editing efficiency of these two loci is 21.5% and 93.3%, respectively (Fig. 4c, d and Additional file 2: Table S4). In plants with a high density of mutations in the promoter region of the *GhTFL1* gene, the individual that was edited at 783 bp upstream of the ATG start codon (*GhTFL1*^pro783^_T0-1) exhibited a phenotype characterized by smaller, darker-colored leaves. Targeted deep sequencing results showed an editing efficiency of up to 87.5% (Fig. 4e and Additional file 2: Table S4). In addition, another plant exhibited a unique growth phenotype with notably larger bracts and sepals as well as multiple flowers clustered together. Following genotyping to determine the present of sgRNAs and editing efficiency showed that this plant carried two sgRNAs simultaneously, and carried both T to C and A to G mutations at 485-504 bp upstream of the ATG translation start site (*GhTFL1*^pro485−504^_T0-1). The editing efficiencies for two loci were found to be 75.6% (T to C at *GhTFL1*^pro485^) and 94.2% (A to G at *GhTFL1*^pro504^), respectively (Fig. 4f and Additional file 2: Table S4). During the flowering period, the *GhTFL1*^pro783^_T0-1 exhibited smaller inflorescences, which resulted in a failure to produce fruit. Consequently, we selected three T0 generation plants (*GhTFL1*^pro783^_T0-1, *GhTFL1*^pro783^_T0-2, and *GhTFL1*^pro783^_T0-3) of *GhTFL1*^pro783^ for the assessment of *GhTFL1* gene expression levels. Additionally, we evaluated the expression levels in T0 generation single plants of *GhTFL1*^pro485−504^ and two T1 generation descendants. The results indicated an increase in *GhTFL1* expression in all three T0 generation *GhTFL1*^pro783^ mutants, with *GhTFL1*^pro783^_T0-1 and *GhTFL1*^pro783^_T0-1 showing a significant upregulation of *GhTFL1* expression compared to the Jin668 control. The *GhTFL1* expression in the T0 generation of *GhTFL1*^pro485−504^_T0-1 was significantly higher than that in the wild type, and this upregulation was also observed in the corresponding T1 generation progeny (Additional file 1: Fig. S11).

**Fig. 5:**
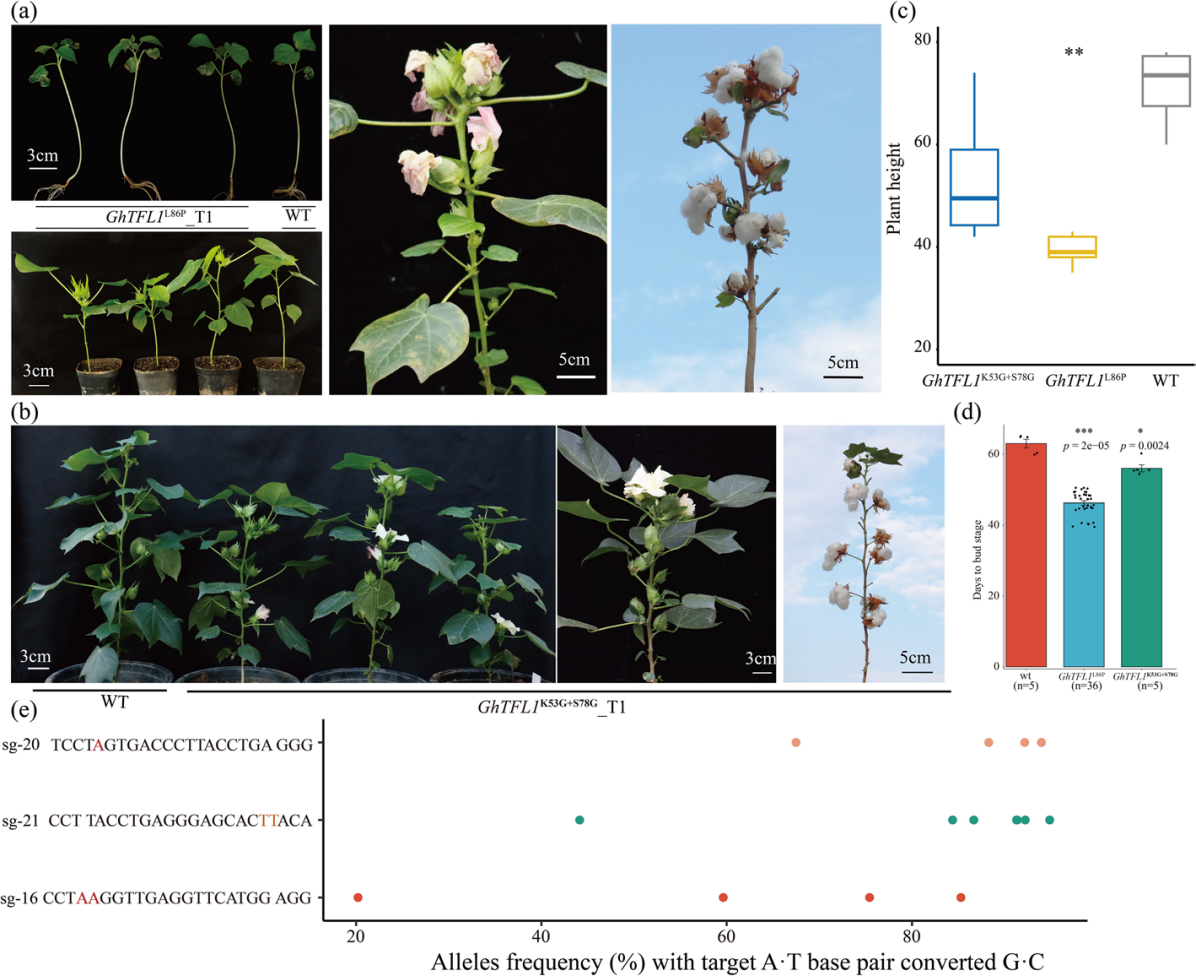
Phenotypic characterization of WT and T1 progenies from *GhTFL1*^L86P^ and *GhTFL1*^K53G+S78G^ mutants. **a** Representative images of WT and T1 progenies of *GhTFL1*^L86P^ at different growth stages. *GhTFL1*^L86P^ plants show the cluster terminal flowers at the top and all axillary meristems subsequently produce floral buds directly on the main stem. **b** Representative images of WT and T1 progenies of *GhTFL1*^K53G+S78G^. The plants show semi-dwarf height, terminating clusters of flowers forming at the tips of the main stems and fruiting branches as monotypic fruiting branches, terminating sympodial growth. **c**,** d** Plant heights (**c**) and days to bud stage (**d**) of WT and the T1 progenies of *GhTFL1*^L86P^ and *GhTFL1*^K53G+S78G^. All data are presented as mean ± sd. *P < 0.05, **P < 0.01, ***P < 0.001 and ****P < 0.0001 by two-tailed Studentʼs *t*-test. **e** The genotype analysis of the T1 progenies of *GhTFL1*^L86P^ and *GhTFL1*^K53G+S78G^. The ordinate represents the target site information, including the 20 bp sgRNA sequence and the adjacent 3 bp PAM sequence, for the T1 generation individual plants of *GhTFL1*^L86P^ and *GhTFL1*^K53G+S78G^. Each dot represents the editing efficiency of an independent sample

High-density mutation of the *GhTFL1* gene showed that mutations at different sites of the *GhTFL1* gene have varying cotton architectures. However, for the purpose of agricultural application, our main interest is the specific phenotype of compact flowering caused by L86P and K53G + S78G base mutation. In order to further investigate the agricultural performance of the L86P and K53G + S78G mutants of GhTFL1, especially their potential to promote compact cotton plant architecture, we planted offspring corresponding to *GhTFL1*^L86P^ and *GhTFL1*^K53G+S78G^ to evaluate plant height and budding stage in greenhouse and farmland. Compared to the wild-type Jin668, the *GhTFL1*^L86P^ mutants entered the budding stage approximately 40 days after emergence and exhibited a terminal flower phenotype, in which both the apical and axillary flowers clustered on the main stem of the plant. Determinate growth of the stem leads to plant dwarfing (Fig. 5a–d). The T1 generation of *GhTFL1*^K53G+S78G^ exhibits a more pronounced phenotype than the T0 generation, with fruit branches that are shortened and terminated in clusters. Additionally, the apical meristems of the T1 generation of *GhTFL1*^K53G+S78G^ maintain indeterminate growth, resulting in a semi-dwarf stature of the plants when compared to the wild-type Jin668 (Fig. 5b–d). At the same time, we also performed surrogate positive identification and targeted deep sequencing on the obtained T1 plants. T-DNA segregation and stable inheritance of A-to-G point mutation were identified in the offspring of L86P mutant (Fig. 5e and Additional file 1: Fig. S12).

### The point mutation of *GhTFL1* changed the spatial structure and prevented the interaction with GhAP1 and Gh14-3-3

**Fig. 6:**
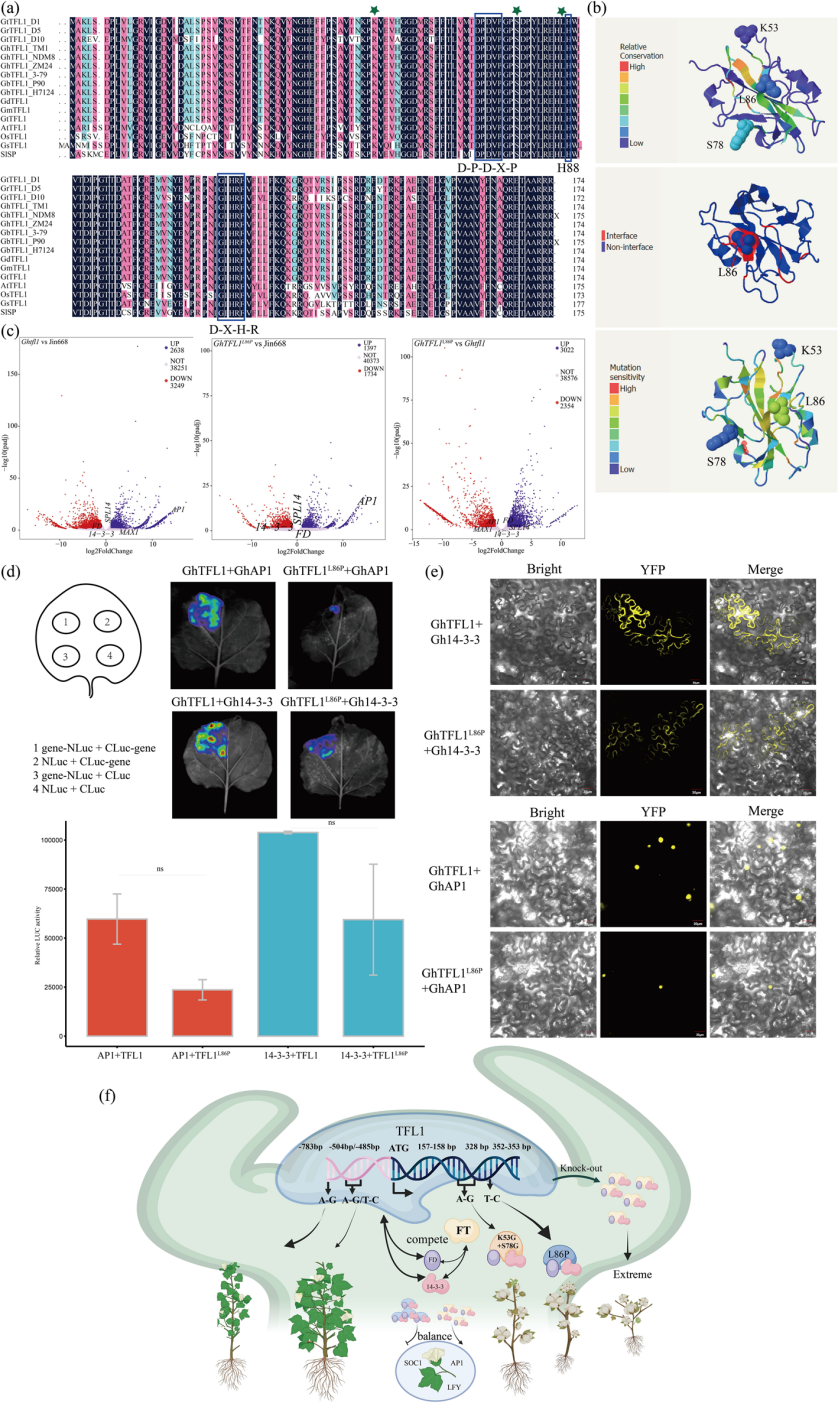
Molecular mechanism of plant architecture in the cotton GhTFL1^L86P^ and GhTFL1^K53G+S78G^ mutants. **a** Comparative amino acid sequences and structural domain analysis of *GhTFL1* from different plants. Three amino acid sites, K53, S78, and L86, are marked with green pentagrams; two structural domains are marked with blue boxes. **b** Analysis of conserved regions, contact interfaces, and mutation-sensitive regions of GhTFL1 protein. **c** Transcriptome analysis of stems and leaves of *Ghtfl1*, *GhTFL1*^L86P^, and Jin668. Volcano plots of differential genes under comparison of the three lines in stems. **d** A firefly LCI assay confirms that GhTFL1 and GhTFL1^L86P^ interact with GhAP1 and Gh14-3-3, respectively. A quantitative comparison of luciferase signals showed that GhTFL1 interacted more strongly with GhAP1 and Gh14-3-3, respectively, than GhTFL1^L86P^ interacted with GhAP1 and Gh14-3–3. **e** Bimolecular fluorescence complementation (BiFC) assay showing that GhTFL1 and GhTFL1^L86P^ interact with GhAP1 in the nucleus and GhTFL1 and GhTFL1^L86P^ interact with Gh14-3-3 in the nucleus and membrane. The N-terminus of yellow fluorescent protein (YFP) was fused to GhTFL1 and GhTFL1^L86P^, while the C-terminus of YFP was fused to GhAP1 and Gh14-3-3. **f** Model for antagonistic roles of TFL1 and FT in promoting branch or floral fate. Schematic model illustrating the role of GhTFL1. During the nutritional growth phase, GhTFL1 binds to Gh14-3-3, GhFD to repress the downstream flowering gene *GhAP1*, thereby maintaining the nutritional growth of the plant. When the *GhTFL1* gene is completely knocked out, nutritional growth is prematurely terminated, resulting in early flowering, reduced plant height, and other phenotypes. When a single base mutation occurs at some of the *GhTFL1* loci, the function of *GhTFL1* to repress downstream flowering genes is diminished, resulting in mutants with intermediate phenotypes. Created with BioRender.com

To elucidate the molecular mechanism of the *GhTFL1*^L86P^ and *GhTFL1*^K53G+S78G^ mutations, the homology of *TFL1* was compared in sequenced diploid, tetraploid, and wild species of cotton, as well as Arabidopsis, soybean, and tomato. The results showed a high degree of similarity in the amino acid sequence of *TFL1* across these various species. The D-P-D-X-P (70-74) and G-X-H-R (115-118) structural domains of the *TFL1* and the key amino acid site Tyr85(Y)/His88(H), which distinguishes the function of FT and TFL1 proteins, are highly conserved across species (Fig. 6a). Further analysis of the amino acid sequence of *GhTFL1* revealed that the *GhTFL1*^L86P^ mutant site is located adjacent to H88, while S78 residue in *GhTFL1*^K53G+S78G^ is positioned three amino acids downstream of the highly conserved structural domain D-P-D-X-P and is also highly conserved in several species. Whereas K53 is conserved in most plant species, the site is R in rice and tomato (Fig. 6a). To better understand the role of L86P as well as K53G + S78G in maintaining GhTFL1 protein function, the 3D structure, function, and mutational sites of the GhTFL1 protein were analyzed using Phyre2 [38]. The predicted amino acid conserved, protein–protein interaction site, and amino acid mutation sensitivity were analyzed and all three sites, L86, K53, and S78, were found to be less conserved. L86 was immediately adjacent to the predicted protein interaction site, H85, and showed strong mutation sensitivity. From the results of the Phyre2 analysis, it was hypothesized that mutation of the L86 site would cause a conformational change in the GhTFL1 binding site, thereby affecting the ability of GhTFL1 to bind to other interacting proteins (Fig. 6b).

To further clarify whether the *GhTFL1*^L86P^ mutation truly affects protein binding capacity and thus causes changes in the expression of upstream and downstream regulatory genes, a transcriptome sequencing analysis was performed using the CRISPR/Cas9 knockout (*Ghtfl1*), GhABE8e base edit (*GhTFL1*^L86P^) and WT plant (Jin668). Transcriptome data was compared among *Ghtfl1*, *GhTFL1*^L86P^, and Jin668, which revealed 12,997 and 5462 differential genes in leaves were identified in *Ghtfl1* VS Jin668 and *GhTFL1*^L86P^ VS Jin668, respectively. In stems, 5887 and 3131 differential genes were identified in *Ghtfl1* VS Jin668 and *GhTFL1*^L86P^ VS Jin668, respectively, while 4248 and 5276 differential genes were identified in leaves and stems, respectively, in the comparative analysis between *Ghtfl1* and *GhTFL1*^L86P^ (Additional file 2: Table S5). Clearly, there was a higher number of differentially expressed genes in the leaves and stems of plants following *Ghtfl1* compared to Jin668 than *GhTFL1*^L86P^ compared to Jin668. This finding is consistent with the truth that the CRISPR/Cas9 knocking out genes resulting in extreme mutant phenotypes, while base editing with *GhTFL1*^L86P^ caused differential expression of a smaller number of genes, suggesting that base editing acts as a fine-tuned approach for influencing gene expression. The further functional annotation of the differentially expressed genes in the stems of *Ghtfl1* and *GhTFL1*^L86P^ revealed that some floral meristem identity, sepal and petal identity genes, such as *APETALA1* (*AP1*), showed significantly up-regulated expression (Fig. 6c).

In *Arabidopsis thaliana*, TFL1 protein can interact with 14-3-3 and FD protein to form an active TFL1-FD-14-3-3 complex to inhibit the expression of *AP1*, thus maintaining nutritional growth. In order to verify whether *GhTFL1*^L86P^ mutation will change the ability of GhTFL1 to bind to other interacting proteins, the interaction of GhTFL1 and mutant GhTFL1^L86P^ proteins with Gh14-3-3 and GhAP1 was analyzed by Yeast two-hybrid (Y2H), firefly LCI and BiFC assays, respectively. The results showed that both GhTFL1 and GhTFL1^L86P^ were able to interact with GhAP1 in the nucleus, but the interaction strength of GhTFL1^L86P^ with the GhAP1 protein was weaker than that of GhTFL1-GhAP1 interaction. It is noteworthy that both GhTFL1 and GhTFL1^L86P^ were found to interact with Gh14-3-3 in the nucleus and cell membrane. Interestingly, our study also revealed differences in the strength of the interaction between GhTFL1 and GhTFL1^L86P^ with Gh14-3-3, as determined through LCI assays (Fig. 6d, e). These results indicate that the GhTFL1^L86P^ mutation only weakens the binding strength of the GhTFL1 to other proteins, but does not lead to a complete loss of GhTFL1 function. This explains why the GhTFL1^L86P^ mutation results in a weak mutant phenotype compared to the Ghtfl1 mutation generated using the CRISPR/Cas9 system.

Taken together, during nutritional growth, GhTFL1 interacts with Gh14-3-3 and GhFD and acts to repress flowering by modulating the expression of the downstream floral meristem (FM) identity gene *AP1*, thereby maintaining nutritional growth. However, when conditions for reproductive growth are reached, the GhFT protein competes with GhTFL1 to interact with Gh14-3-3 and GhFD forming a tripartite complex referred to as the florigen activation complex (FAC) and activated *AP1*. However, when *GhTFL1* is absent, the timing of the transition between nutritional growth and reproductive growth is disrupted, causing the phenotype of early flowering and production of terminal flowers on both main stems and branches in cotton. When base mutations occurred at different loci of *GhTFL1*, cotton showed phenotypes with varying degrees of architecture variation (Fig. 6f).

## Discussion

The florigenin (FT) and anti-florigenin (TFL1) hormone system plays a significant role in regulating plant architecture by synergistically controlling nutritional and reproductive growth [39–41]. Genetic improvement of plant architecture has been successfully driven by CRISPR/Cas9-targeted regulation of the FT-TFL1 system in the tomato [2, 42, 43]. However, the application of the FT-TFL1 system in cotton breeding has not yet evaluated. Mutations of *GhTFL1* in cotton have a general dosage sensitivity, making it difficult for conventional breeding to achieve improvements in plant architecture in the short term [44]. Here, the knockout of the *GhTFL1* gene by CRISPR/Cas9 has resulted in an early flowering phenotype with extreme dwarfism mutants that are unsuitable for production applications. By contrast, base editing provides an efficient, precise means to achieve base mutations in target genes without creating DNA double-strand breaks nor requiring the addition of DNA repair templates. Therefore, base editing, dual-base editor in particular, has become an important means of directed evolution of endogenous functional genes, discovery of new beneficial variants, and fine-tuning of gene expression [5, 45, 46].

Upland cotton is an allotetraploid (AtAtDtDt) species with a complex and large genome [25]. Although advances in gene editing have been made with CRISPR/Cas9 [47], CRISPR/Cpf1 [26], CRISPR/C2c1 [48], CBE [17], and ABE [16], these systems especially base editing still need to be further developed and optimized compared to simple genomic crops that have shown good editing efficiency. In order to rapidly identify the function of nucleotide variants in the *GhTFL1* gene and identify agronomically beneficial mutants, we developed an advanced cotton-compatible ABE vector (GhABE8e) with optimized adenosine deaminase TadA8e (V106W). GhABE8e exhibits an enhanced base editing activity in cotton, almost 99.9%, indicating the improved applicability of TadA8e relative to TadA7.10 for gene editing in cotton. Notably, GhABE8e showed robust mutagenesis at two or three target sites using multiple sgRNAs simultaneously, which is especially valuable for the artificial evolution of functional proteins through base editing with a limited number of sgRNAs against the target region. Furthermore, GhABE8e exhibits a broader editing window than GhABE7.10n (that displayed activity only at Position 5 of sgRNA2) [16] and extends to A4–A12 in cotton, more importantly, GhABE8e does not generate sgRNA-dependent off-target effect at both DNA and RNA levels.

In addition to the editing window limiting in range and number of targets, the requirement for a PAM with an NGG on the 3′ end of the protospacer also is a severe limitation on the design of sgRNAs [49]. To break the limitations of PAM, engineered Cas9 variants and new types of Cas protein with altered PAM sequences have been used in base editors [50–52]. Previous studies have shown that the dLbCpf1-mediated CBE system that recognizes PAM as TTTV can work in human cells [35, 53]. Given this, we successfully applied ABE7.10-dCpf1 fused to TadA7.10 and dLbCpf1 for the first time in plants, despite exhibiting a low editing efficiency compared to GhABE7.10-nCas9 [16]. Here, we further devised and evaluated dLbCpf1-mediated ABE tool that fused dLbCpf1 and TadA8e in cotton. GhABE8e-dCpf1 exhibited high editing efficiency compared to GhABE7.10dCpf1, which indicates that TadA8e is more compatible with various Cas proteins than TadA7.10, and also more compatible with Cas9 variants compared to Cpf1.

The adverse effects of off-target mutations on plant functional genomics research and molecular breeding practices based on genome editing strategies cannot be overlooked. Studies in rice have detected A-to-G off-target mutations in single plants edited with SpCas9n-TadA8e, and have found a correlation between the off-target effects of ABE8e and its expression levels [36]. Additionally, using rice as a model for deciphering the off-target effects of plant genome editing mediated by the PAM-relaxed adenine base editor (nSpRY-ABE8e), it was observed that nSpRY-ABE8e-edited rice plants produced ABE8e-dependent off-target mutations at the genome-wide level [54]. In our study, we conducted a comprehensive assessment of the specificity of GhABE8e (V106W) at both DNA and RNA levels in allopolyploid cotton using deeply sequenced genomes and transcriptomes. Our results revealed that GhABE8e did not induce sgRNA-dependent off-target effects at either the DNA or RNA level. Furthermore, the optimized deaminase TadA8e (V106W) used in GhABE8e did not exhibit sgRNA-independent off-target effects at the DNA level. Regarding RNA-level sgRNA-independent off-target effects, our analysis indicated that A-to-G off-target mutations induced by deaminases TadA8e accounted for less than 1% of all single nucleotide variants detected in the transcriptome, which differs from the results observed with ABE8e in rice. The differences in specificity between ABE8e in the allopolyploid cotton genome and the diploid rice genome may be attributed to the complexity of the cotton genome, which contains a large number of repetitive sequences and homologous genes, potentially increasing the number of target sites and enhancing the action of GhABE8e. Additionally, the chromatin structure and epigenetic features of the cotton genome, such as DNA methylation and histone modifications, may influence the specificity of the editing tool. Furthermore, the process of tissue culture may also affect the specificity of genome editing, as tissue culture conditions can induce changes in the physiological state and gene expression patterns of cells, potentially influencing the interaction between the editing tool and its targets. It is important to note that non-specific mutations have relatively limited negative effects in plant breeding, and base mutations at the RNA level are not stably inherited in plants. Therefore, a higher tolerance for off-target effects of ABE8e in plants may be warranted.

Using the newly developed efficient base editing tool for cotton, we have explored unprecedented applications of base editing in the study of the genetic regulatory module of GhTFL1. We have implemented artificial evolution of the cotton *GhTFL1* gene by employing GhABE8e which resulted in a series of diverse weak alleles of the *GhTFL1* through high-density point mutations. Meanwhile, differences in *GhTFL1* gene function caused by mutations in the coding region were also explored by base editing at different loci. The ability to effectively edit gene promoters, thereby altering the expression levels or patterns of target genes, is emerging as a critical tool for elucidating the biological functions of these promoters. Moreover, the creation of promoter-edited germplasm with valuable traits for breeding is becoming an increasingly important area of focus. Recent advancements have demonstrated that targeted editing of gene promoter regions using Cas9 and Cas12a genome editing technologies can enhance or improve crop agronomic traits without adversely affecting other characteristics [55–58]. In light of these developments, our study employed GhABE8e to introduce point mutations into the promoter region of the *GhTFL1* gene, leading to observable phenotypic changes. Although the constraints of GhABE8e’s PAM specificity and mutation types limited our ability to generate a broad spectrum of continuous phenotypes, our findings provide preliminary evidence supporting the feasibility of using base editing tools for promoter region modifications. This approach holds promise for refining gene expression in a precise and controlled manner, which is essential for both fundamental research and the development of improved crop varieties.

## Conclusions

In summary, we have established and optimized multiple base editors for cotton and utilized them to carry out high-density base mutations in *GhTFL1*, from which new genetic loci were identified, creating new cotton germplasms with moderate height, shortened fruiting branches, compact architecture and shortened fertility, and rapidly achieving targeted evolution of the *GhTFL1* gene. The rapid evolution of the *GhTFL1* gene through base editing will be of great value for the improvement of cotton easy for mechanical harvesting and also opens up new avenues for the directed evolution of the coding and promoter regions of the cotton gene to obtain weak mutants required for remodeling agronomic traits.

## Methods

### Plasmid vectors construction

GhABE8e plasmid vectors were modified from the GhABE7.10 generated in our previous report [16]. TadA-TadA7.10 in GhABE7.10 were deleted by double digestion of SalI (NEB) and Bsu36I (NEB) to obtain the backbone of GhU6-7: gRNA-Ubi: Cas9n (D10A). The amino acid sequences encoding TadA8e (V106W), bpNLS, and the linker peptide [19] were codon-optimized for expression in cotton and the corresponding 651-bp nucleotides were synthesized by GenScript (Nanjing, China). This synthetic nucleic acid sequence was inserted into the backbone of GhU6-7: gRNA-UBi: Cas9n (D10A) to generate G. hirsutum-Adenine Base Editor 8e (GhABE8e). GhABE8e-dCpf1 plasmid vectors were modified from the GhABE8e (Additional file 3: Sequences). The dCpf1 was amplified from our previous vector GhABE7.10dCpf1 with the primer pair dCpf1-F/dCpf1-R and cloned into the GhABE8e vector from which nCas9 was deleted by double digestion (Additional file 2: Table S6).

To explore GhABE8e and different ABE variants derived from TadA8e with new properties in cotton, 13 targets (sgRNA1-sgRNA13) located at 3 genes (Additional file 2: Table S1) were selected from previous studies using GhABE7.10n or using the online webtool CRISPR-GE designed by us. Among these targets, sgRNA1 and sgRNA2 were used to test the editing efficiency of GhABE8e compared with GhABE7.10n. sgRNA3-sgRNA7 in tandem and were used to evaluate the efficiency of simultaneous editing by GhABE8e at multiple loci. sgRNA8-sgRNA11 sites with different A-Base distributions were used to evaluate the editing window of ABE8e. sgRNA12 sites with TTTV-PAM were used to determine the editing efficiency of GhABE8e-dCpf1. sgRNA13 to determine the editing accuracy of GhABE8e. All sgRNA expression cassettes were PCR amplified from PGTR plasmid and inserted into the corresponding binary plasmids using the ClonExpressII One Step Cloning Kit (Vazyme, Nanjing, China) and were expressed driven by the cotton endogenous U6 promoter according to our previous publication (Additional file 2: Table S7) [59–61].

### *Agrobacterium*-mediated cotton transformation

The *Gossypium hirsutum* genotype Jin668 was used in this study [62]. All constructs were introduced into *Agrobacterium* strain GV3101 (kanamycin as a selectable marker) via electroporation, and then the *Agrobacterium*-mediated transformation was performed following a protocol mentioned previously [16, 63].

### On-target mutation detection in transgenic cotton

The cotton genomic DNA was extracted from T0 and T1 individual regenerated and controlled cotton plants by the cetyltrimethylammonium bromide method, which were used as the template for PCR amplification. PCR was performed by specific primers (Additional file 2: Table S8) to amplify the nCas9 and sgRNA sequence region of the binary vectors to confirm transgenics. The genomic regions spanning the target sites were PCR amplified using target-specific primers with unique barcode tags consisting of six bases. The resulting PCR products (~ 220–230 bp) were mixed in equal amounts to construct a sample library and then were purified using a PCR Purification Kit (OMEGA, D2500-02). The purified sample library was sequenced on an Illumina HiSeq 2500 sequencer following the manufacturer’s protocol (Illumina, San Diego, CA) and analyzed with CRISPResso2 for detecting potential mutations. In addition, wild-type (WT) plants were used to filter out background mutations in the cotton population. Control plants (Negative) were used to evaluate the mutations occurring during tissue culture and transformation.

### Detection of off-target mutations by genome and transcriptome sequencing analysis

Genomic DNA and RNA wereas extracted from four samples including an edited plant generated by the GhABE8e with one sgRNA, a plant carrying the GhABE8e without sgRNA, a negative plant (following tissue culture and plant generation but without T-DNA insertion), and a wild type (Jin668) as controls as previously described. The Genomic DNA and RNA of four plant samples were sequenced using the Illumina HiSeq X Ten platform in accordance with the manufacturer’s recommendations (Illumina, San Diego, CA), ultimately resulting in more than 1 Tb WGS data and 90 Gb transcriptome raw reads (the average depth being 50 ×), respectively. The data of WGS and transcriptome sequencing was analyzed according to our previous publication [16].

### sgRNA library design and assembly for evolving *GhTFL1*

The BEsgRNADe algorithm was developed in python, and the web portal was implemented with the Django framework. The genomic DNA sequence of *GhTFL1* (*Ghir_D07G011770.1*) was retrieved from the *Gossypium hirsutum* TM-1 genome and verified through searching sequence of the Jin668 genome. The 525-bp open reading frame sequence was submitted to the online tool BEsgRNADe. Targets with adenine candidates available and amino acid changes in the editing window were identified by selecting the BEsgRNADe ABE target design function. In order to further ensure that diverse phenotypic are obtained, online tools Phyre2 [38] for protein structure, function, and variant prediction analysis were used to screen these targets resulting in a total of 17 sgRNAs.

In order to fine-tune the expression of the gene and obtain the weak mutant required for agricultural production, we performed gene editing modification on the promoter region of *GhTFL1*. The 800-bp promoter sequence of *GhTFL1* (*Ghir_D07G011770.1*) was obtained from the *Gossypium hirsutum* TM-1 genome and verified through searching sequence of the Jin668 genome. Combined with the cis-regulatory elements on the promoter predicted by PlantCARE [64] online tool, 9 sgRNAs were designed.

In the promoter region, among the 9 sgRNAs (sg-1 to sg-9), sg-1 was constructed into the ABE8e vector as a single-target structure, while sg-2 and sg-3, sg-4 and sg-5, sg-6 and sg-7, and sg-8 and sg-9 were constructed into the GhABE8e vector as double targets. In the CDS region, out of the 17 sgRNAs (sg-10 to sg-26), sg-16 and sg-20 were combined as a double target, and sg-23, sg-24, sg-25 were combined as a set of three targets in one GhABE8e vector, with the rest constructed as single targets. In total, 26 sgRNAs were designed for both the promoter and CDS sequences, resulting in 19 vectors after combination (Additional file 2: Table S9). All 26 sgRNA expression cassettes were PCR amplified from the PGTR plasmid and inserted into the GhABE8e binary plasmids using the ClonExpressII One Step Cloning Kit (Vazyme, Nanjing, China). The 19 constructed vectors were transferred to *Agrobacterium* strains using the electroporation method. Subsequently, these 19 *Agrobacterium* strains containing the GhABE8e vector were individually used to infect cotton hypocotyls in sequence.

More than 200 independent transgenic T0 plants were obtained, which were generated through *Agrobacterium*-mediated transformation with 26 GhABE8e as described previously. The primers used in this study are listed in Additional file 2: Table S6.

### RNA-seq analysis

The RNA sequencing reads from different tissues of Jin668, *GhTFL1* knockout (*Ghtfl1*) and GhABE8e base edited *GhTFL1* (*GhTFL1*^L86P^) were removed of adapters and trimmed for low-quality bases using Trimmomatic (v.0.39) [65]. The clean reads were then mapped to the cotton genome using HISAT2 (v.2.2.1) [66] with default parameters. The expression level (transcripts per million; TPM) of genes was calculated by StringTie (v.2.1.4) [67]. A gene was considered to be expressed if its TPM > 0. Subsequently, differentially expressed genes were identified by using the DESeq2 [68] package with at least a twofold change in expression and a false detection rate (FDR) value of less than 0.05.

### BiFC and LCI assay

For LCI assays, the CDSs of *GhTFL1* and *GhTFL1*^L86P^ were constructed on the JW771 vector, and the CDSs of *Gh14-3-3* and *GhAP1* were constructed on the JW772 vector, respectively. For BiFC assays, the CDSs of *GhTFL1* and *GhTFL1*^L86P^ were constructed on the pXY104 vector, and the CDSs of *Gh14-3-3* and *GhAP1* were constructed on the pXY106 vector, respectively. All vectors were transformed into *Agrobacterium tumefaciens* strain GV3101 that were infiltrated into young leaves of *Nicotiana benthamiana*. Fluorescence signals of LUC luminescence in LCI and YFP fluorescent proteins in BiFC assays were observed by a cryogenically cooled CCD camera (Lumazome PyLoN 2048B) and a confocal microscope (Olympus FV1200) respectively as described previously [69]. The primers used in this study are listed in Additional file 2: Table S6.

### RNA isolation and qRT-PCR

Leaf tissue of cotton was ground in liquid nitrogen and total RNA was extracted using a Spectrum™ Plant Total RNA Kit (STRN250; Sigma, St.Louis, MO, USA) according to the manufacturer’s instruction. RNA was quantified by Nanodrop One spectrophotometer (Thermo Scientific), normalized and cDNA synthesis was performed with SuperScript III Reverse Transcriptase (Thermo Fischer Scientific). The expression levels were measured by real-time qPCR using Universal SybrGreen Master Mix (Bio-Rad) on the CFX96 Real-Time System (Bio-Rad). The housekeeping genes GhUBQ7 (GenBank: DQ116441.1) were used as the internal controls for cotton. The primers used are listed in Additional file 2: Table S10. Three technical replicates and a Two-Step RT-PCR method were performed for each experiment. The relative quantification analysis was calculated by using 2^−ΔΔCT^. Error bars represent the standard deviation.

### Statistical analysis

R (v.4.0.0; https://www.r-project.org/) software was used to analyze the data. All numerical values are presented as mean ± sd. Differences between control and treatments were tested using two-tailed Student’s* t*-tests. The threshold for significant was set to *P*-value < 0.05.

### Supplementary Information

**Additional file 1.** Supplementary figures S1-S12.

**Additional file 2:** **Table S1.** Summary of editing targets in this study. **Table S2.** Summary of genome-wide potential off-targets predictions by Cas-OFFinder tools for target sgRNA13. **Table S3.** Statistics of mutant genotypes and mutation sites resulting from GhABE8e-mediated base editing in GhTFL1 lines. **Table S4.** GhABE8e-mediated base editing in GhTFL1 T0 lines. **Table S5.** The number of differential genes identified in the three materials. **Table S6.** Primers used for vectors construction and positive test. **Table S7.** Primers used for amplification of target sites in this study. **Table S8.** Primers used for amplicon deep sequencing of target sites in this study. **Table S9.** The 26 targets information of the 19 vectors. **Table S10.** Primers used for qPCR analysis in this study.

**Additional file 3.** Sequence information of the key elements of base editors (TadA8e-nCas9 and TadA8e-dCpf1) tested in this study.

**Additional file 4.** Review history.

## Data Availability

All the sequencing data have been deposited in the National Center for Biotechnology Information (NCBI) Sequence Read Archive (SRA) under project accession numbers PRJNA869341 [70] and PRJNA869343 [71].
