# Supplementary material for: Precise fine-turning of GhTFL1 by base editing tools defines ideal cotton plant architecture
Source: Genome Biol. 2024 Feb 26;25:59. doi: 10.1186/s13059-024-03189-8 (PMC10895741; doi:10.1186/s13059-024-03189-8)
Supplement: Supplementary file 1 — Additional file 1. Supplementary figures S1-S12. [file 13059_2024_3189_MOESM1_ESM.docx]

**Supplementary Materials for**

**Precise fine-turning of *GhTFL1* by** **base editing tools defines** **ideal** **cotton plant architecture**

Guanying Wang^1^, Fuqiu Wang^1^, Zhongping Xu^1^, Ying Wang^1^, Can Zhang^1^, Yi Zhou^1^, Fengjiao Hui^1^, Xiyan Yang^1^, Xinhui Nie^2*^, Xianlong Zhang^1*^, Shuangxia Jin^1*^

^1^ National Key Laboratory of Crop Genetic Improvement, Hubei Hongshan Laboratory, Huazhong Agricultural University, Wuhan, Hubei 430070, China;

^2^ Key Laboratory of Oasis Ecology Agricultural of Xinjiang Production and Construction Corps, Agricultural College, Shihezi University, Shihezi, Xinjiang, 832003, China;

^*^ Corresponding author: [jsx@mail.hzau.edu.cn](mailto:jsx@mail.hzau.edu.cn); [xlzhang@mail.hzau.edu.cn](mailto:xlzhang@mail.hzau.edu.cn); [xjnxh2004130@126.com](mailto:xjnxh2004130@126.com)

# Additional file 1: Figures


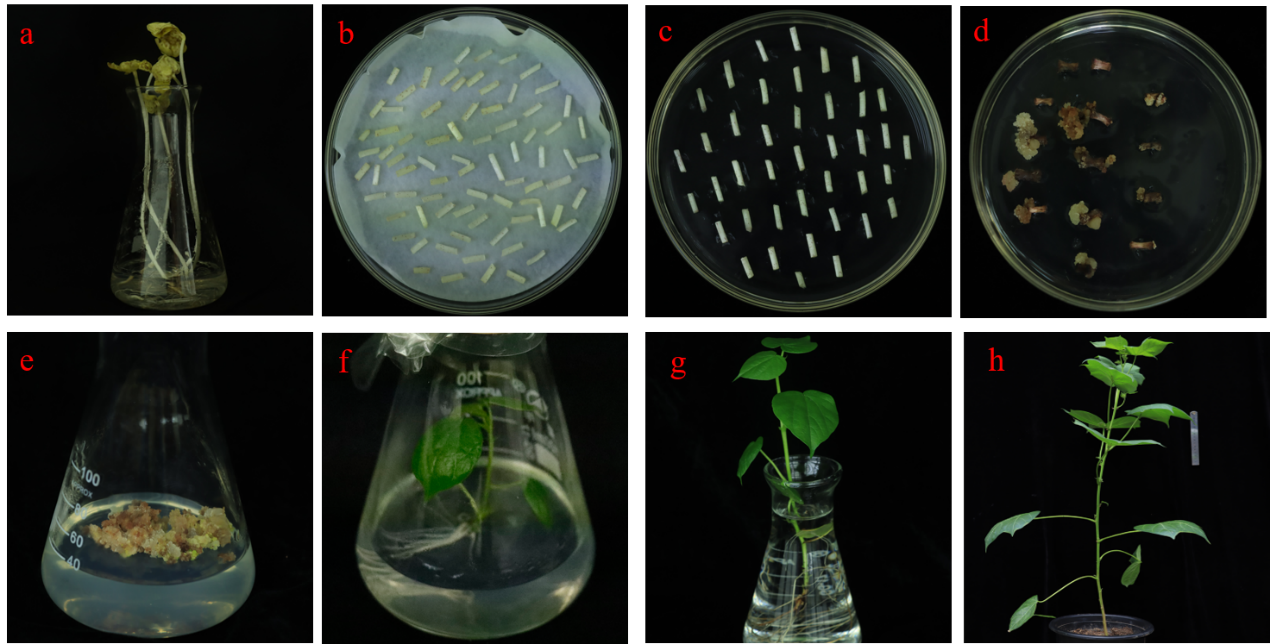


**Fig. S1**. ***Agrobacterium*-mediated genetic transformation and plant regeneration of transgenic plants.** (a) Sterile seedling culture. (b) Agrobacterium Co-culture stage. (c-e) Callus induction and differentiation. (f) Plant regeneration. (g) The acclimatization of regenerated plant in nutrient solution. (h) Transgenic plants grown in the greenhouse.

**
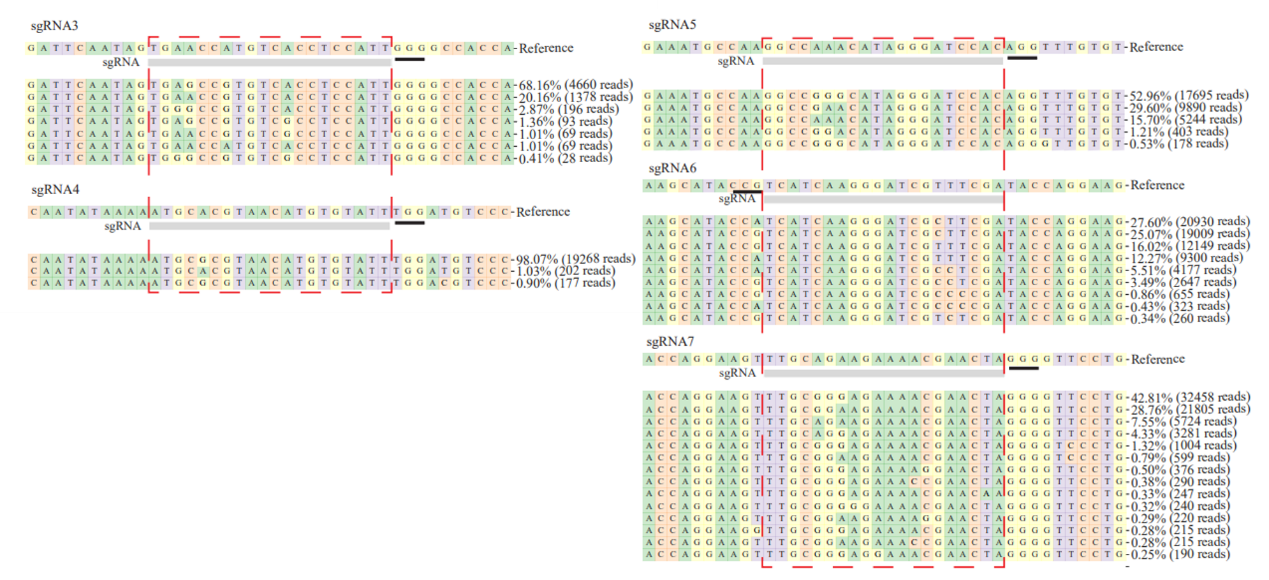
**

**Fig. S2**. **Mutant allele compositions of five target sites edited by GhABE8e in five represented transgenic cotton.** Dashed-line box in red marks the target sequence and the PAM is underlined. Data are collected from one biological replicate in Fig. 1e.


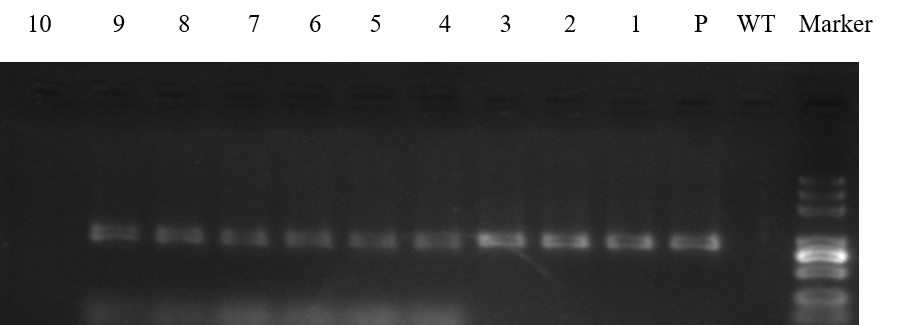


**Fig. S3. Positive identification of T1 generation material by PCR.** Detection of Cas9 in plants. WT, Wild-type, negative control; P, indicates positive plasmid control; 1-10 means T1 line 8_T1_1, 8_T1_2, 8_T1_3, 8_T1_4, 8_T1_5, 11_T1_1, 11_T1_2, 11_T1_3, 11_T1_4 and 11_T1_5, respectively.


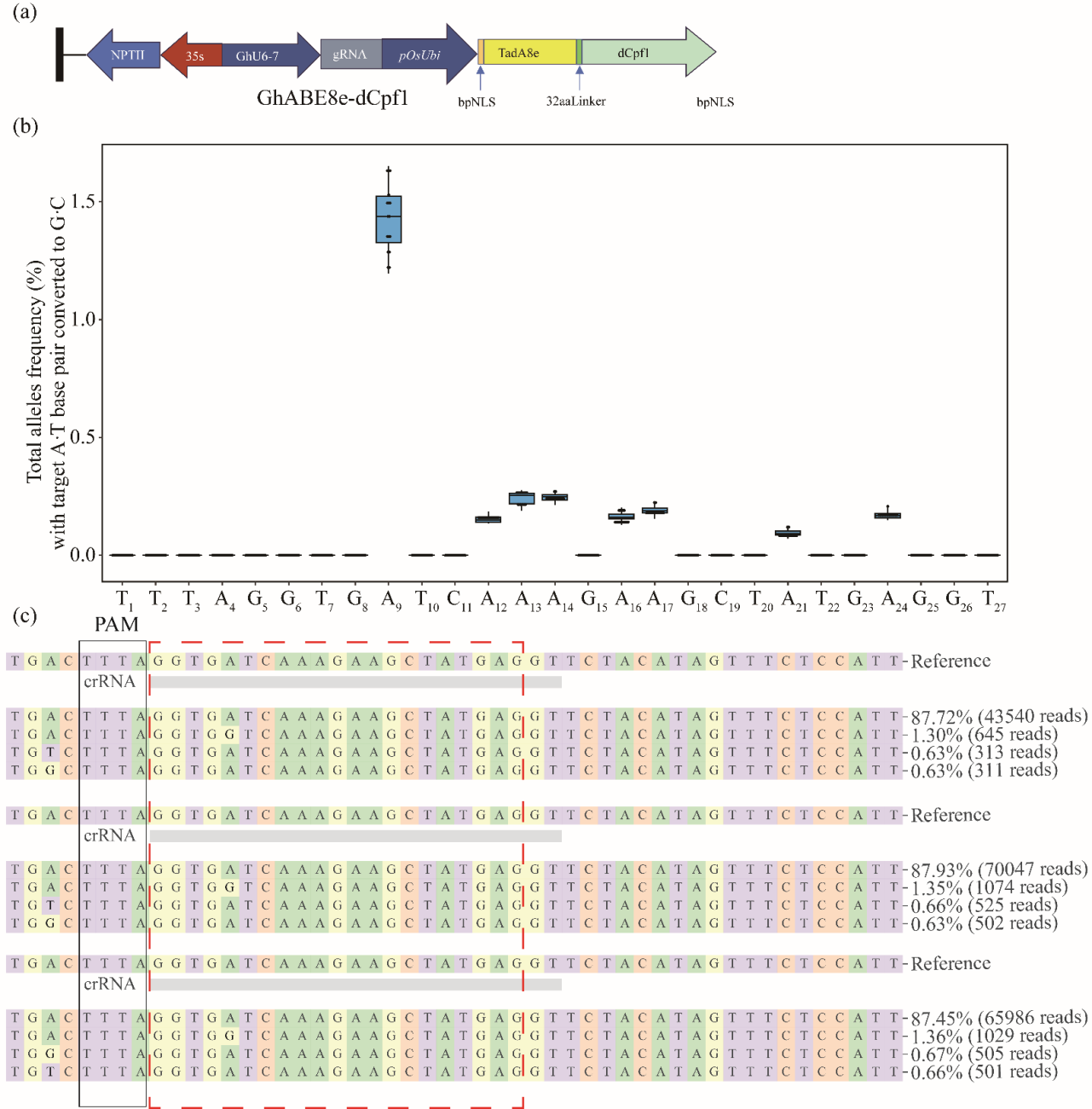


**Fig. S4**. **GhABE8e-dCpf1 mediates A-to-G conversions at crRNA12 as revealed by amplicon deep sequencing.** (a) Schematic diagram of the vector element of dCpf1-fused TadA8e. There are 32 amino acids linker between dCpf1 and TadA8e. dCpf1, catalytically dead *Lachnospiraceae bacterium* Cpf1. (b) GhABE8e-dCpf1 mediates A-to-G conversions at crRNA12 as revealed by amplicon deep sequencing. The middle line of the box represents the median and the bottom and top lines of the box represent the upper and lower quadrilles of the data, respectively. Tail extends to minimum and maximum of data. (c) Mutant allele compositions of three represented transgenic cotton edited by GhABE8e-dCpf1. Dashed-line box in red marks the target sequence and solid-line box in black marks PAM TTTV. Data are collected from one biological replicate in Supplementary Fig. 4b.

**Fig. S5. Expression of genes containing overlapping off-target RNA-SNVs and random simulated genes induced with GhABE8e.** Two-sided unpaired t-test.


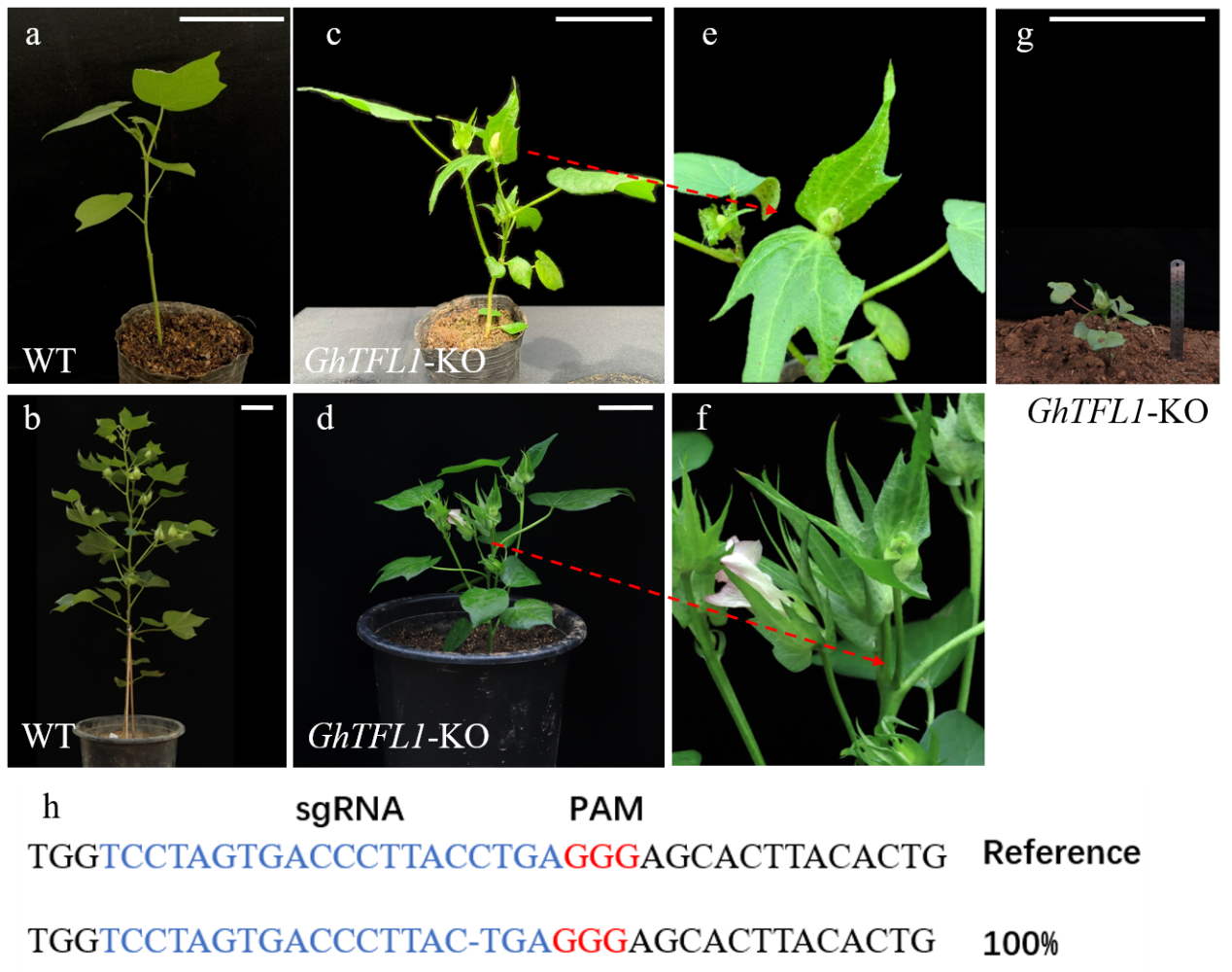


**Fig. S6. Creating extreme dwarf and early flowering cotton using CRISPR/Cas9 knockout of *GhTFL1*.** (a and b) The wild-type at different periods; (c-g) The knockout line. GhTFL1-KO showed an extremely dwarf phenotype, with the apical leaf of the main stem being replaced by a flower bud as an apical flower, while the axillary flower grew directly under the axillary part of the main stem leaf. Scale bars, 10 cm.


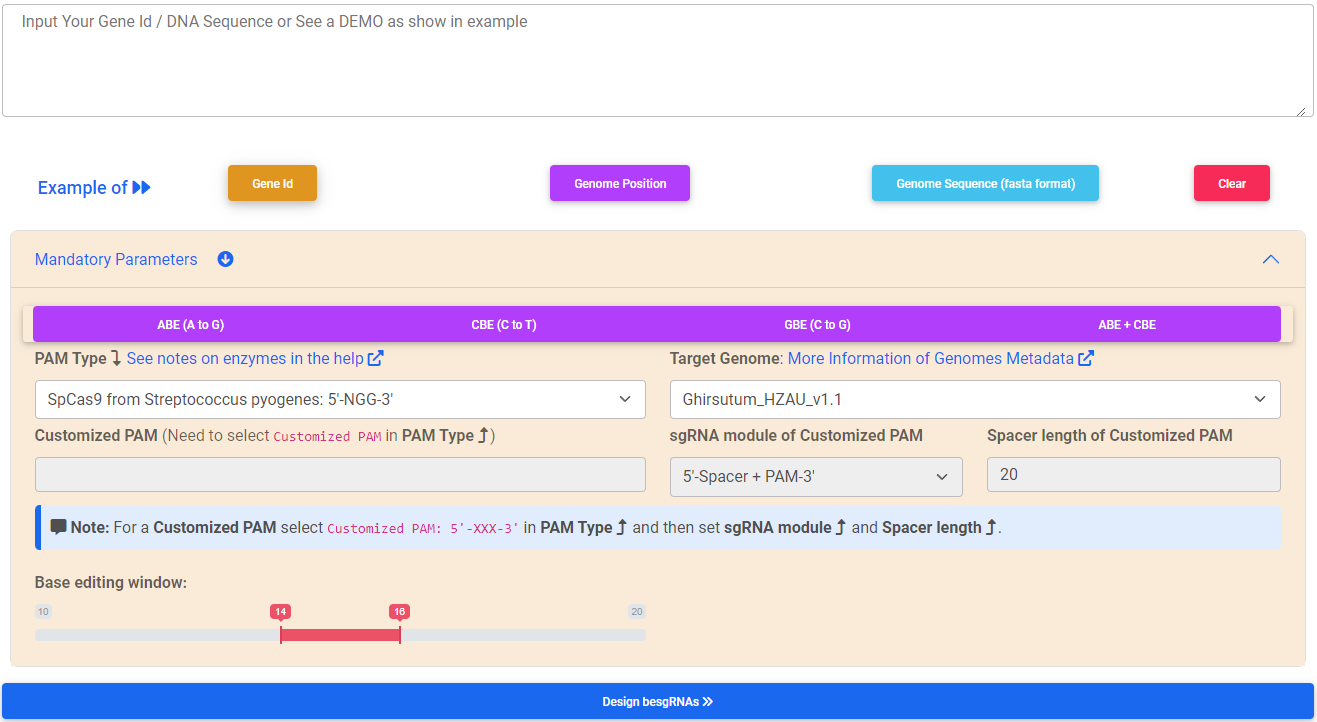


**Fig. S7. Submission page of BEsgRNADe.** Settings panel for reference genome, PAM type, base editing type, and editing window.


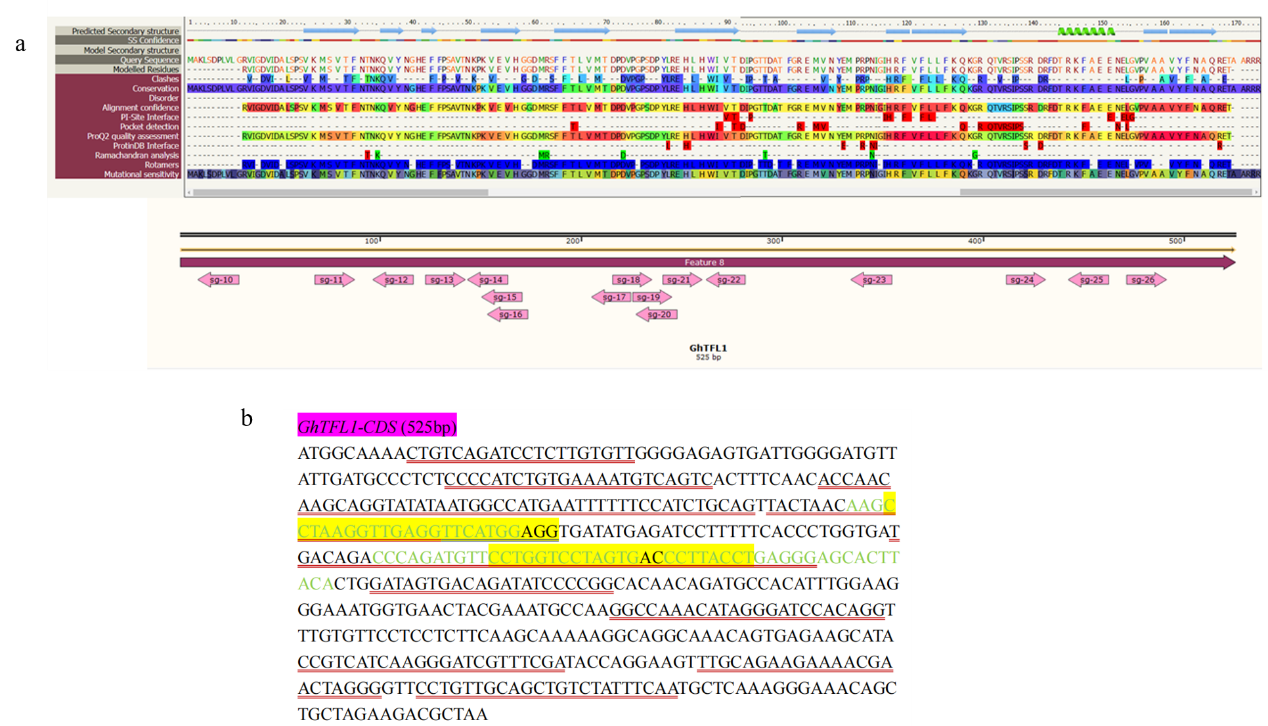


**Fig. S8. The CDS DNA Sequence and sgRNA-Targeting Sites with PAM of GhTFL1 in cotton.** (a) TFL1 protein structural domain analysis using phyre2 and targets distribution; (b) Target sequence distribution, reverse sgRNAs are indicated by red double lines, positive sgRNAs are indicated by green double horizontal lines, and overlapping sgRNAs are distinguished by yellow**.**


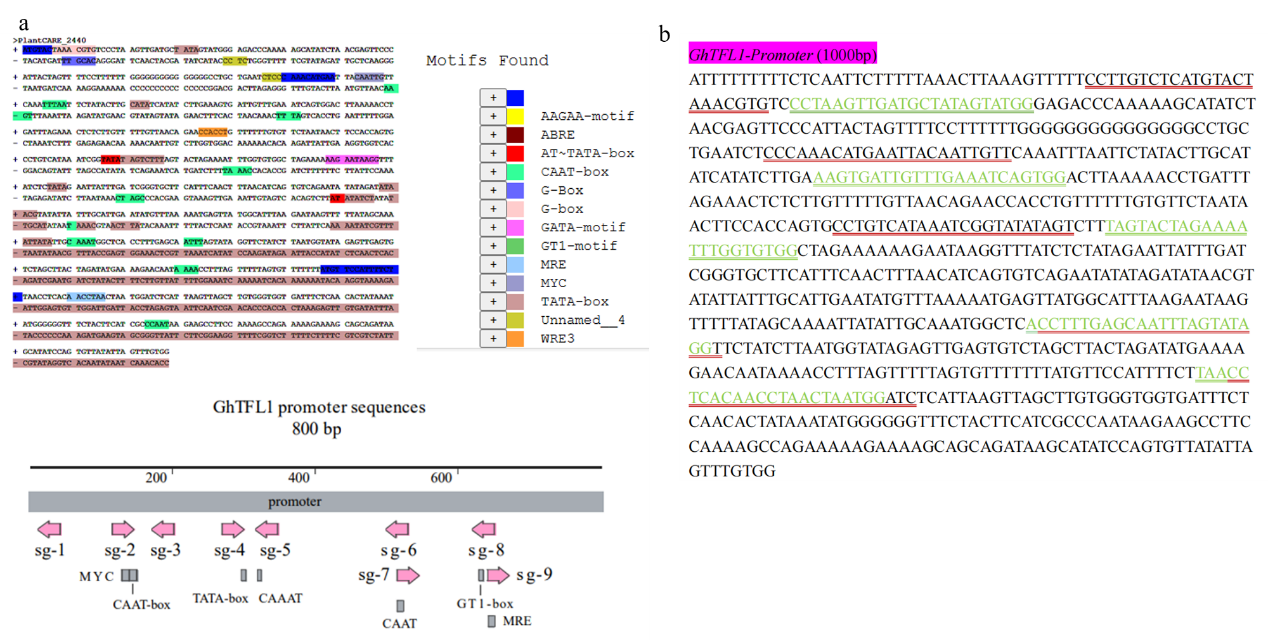


**Fig. S9. The Promoter and genomic DNA Sequence and sgRNA-Targeting Sites with PAM of GhTFL1 in cotton.** (a) Motifs analysis of the GhTFL1 promoter using PlantCARE and targets distribution; (b) Target sequence distribution, reverse sgRNAs are indicated by red double lines, positive sgRNAs are indicated by green double horizontal lines**.**


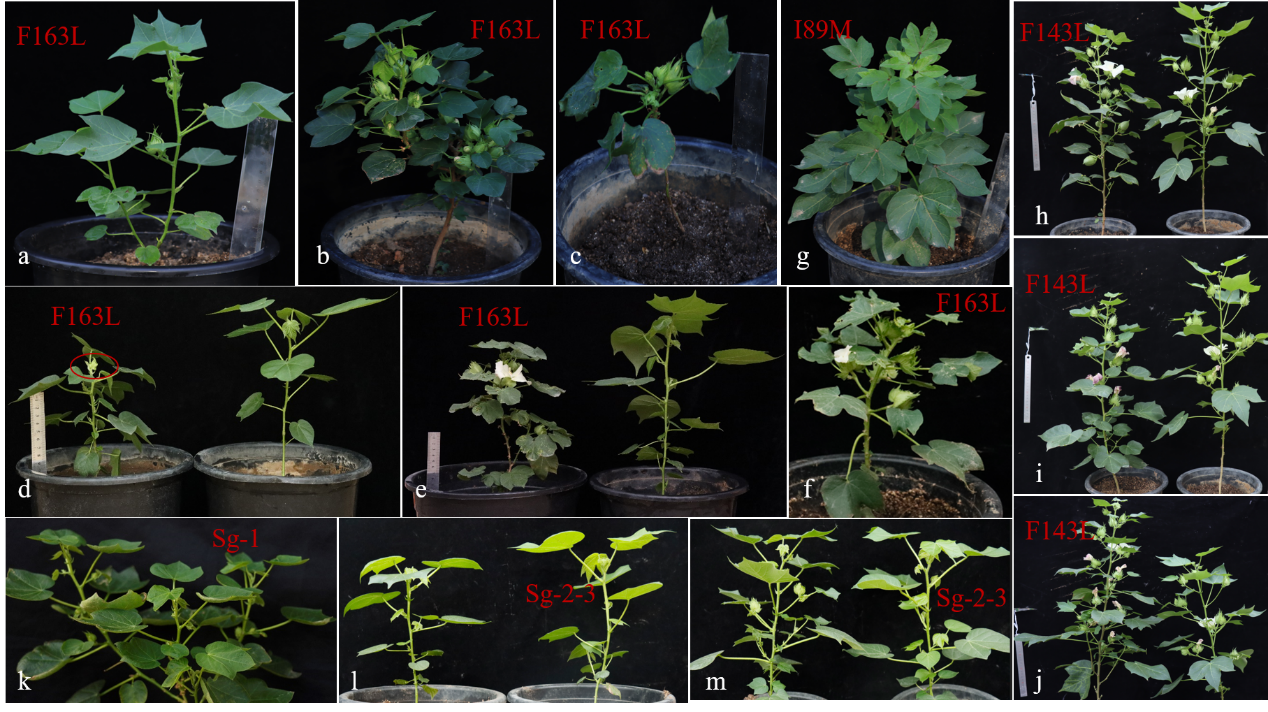


**Fig. S10.** **Different cotton architecture obtained from directed evolution *GhTFL1* by GhABE8e.** (a-f) The phenotypes resulting from the mutation of the 163rd amino acid F to L in GhTFL1, showing dwarf plants with clustered flower buds at the shoot apex; (g) The phenotype after the mutation of the 89th amino acid I to M in GhTFL1, with increased plant branching and changes in leaf morphology, including deeper leaf margin indentations; (h-j) The phenotypes of plants with the 143rd amino acid F mutated to L in GhTFL1, where plants exhibit shortened fruiting branches compared to the wild type; (k-m) The phenotypes resulting from point mutations in the GhTFL1 promoter, with plants carrying mutations at positions 783 upstream of the ATG (sg-1) showing darker and thicker leaves; plants with mutations at positions 663 and 618 in the promoter exhibit a significant delay in flowering.

**
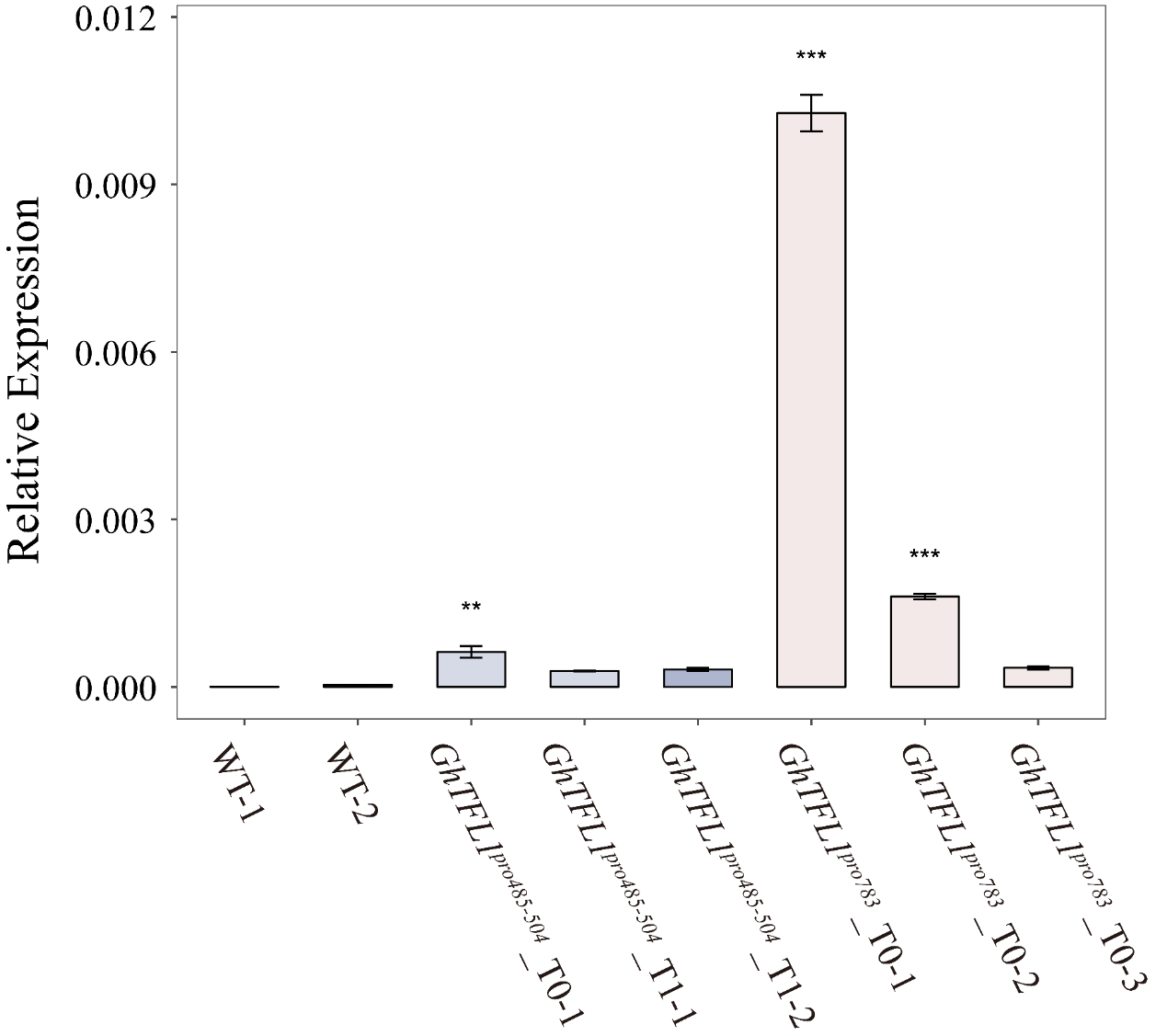
**

**Fig. S11**. Relative expression of GhTFL1 at the RNA level in WT, the T0 and the T1 prohenies from the promoter mutant of GhTFL1. *GhUBQ* was used as internal control.


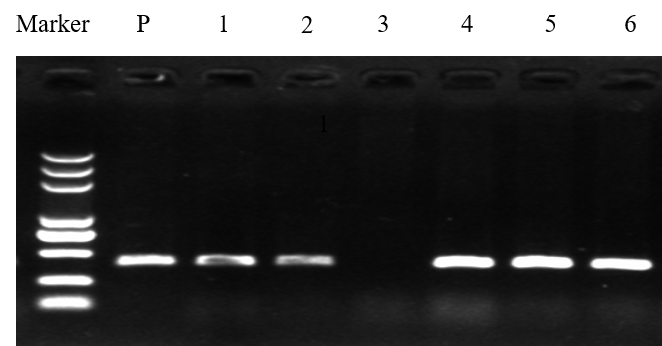


**Fig. S12**. **Positive identification of T1 generation material**. P: positive plasmid control; 1-6 are T1-generation regenerated plants of *GhTFL1*^L86P^, respectively.

# Additional file 2: Tables

**Table S1.** Summary of editing targets in this study.

**Table S2.** Summary of genome-wide potential off-targets predictions by Cas-OFFinder tools for target sgRNA13.

**Table S3.** Statistics of mutant genotypes and mutation sites resulting from GhABE8e-mediated base editing in GhTFL1 lines.

**Table S4.** GhABE8e-mediated base editing in GhTFL1 T0 lines.

**Table S5.** The number of differential genes identified in the three materials.

**Table S6.** Primers used for vectors construction and positive test.

**Table S7.** Primers used for amplification of target sites in this study.

**Table S8.** Primers used for amplicon deep sequencing of target sites in this study.

**Table S9.** The 26 targets information of the 19 vectors.

**Table S10.** Primers used for qPCR analysis in this study.
