# Supplementary material for: Precise fine-turning of GhTFL1 by base editing tools defines ideal cotton plant architecture
Source: Genome Biol. 2024 Feb 26;25:59. doi: 10.1186/s13059-024-03189-8 (PMC10895741; doi:10.1186/s13059-024-03189-8)
Supplement: Supplementary file 3 — Additional file 3. Sequence information of the key elements of base editors (TadA8e-nCas9 and TadA8e-dCpf1) tested in this study. [file 13059_2024_3189_MOESM3_ESM.docx]

# Additional file 3: Supplementary amino acid sequences of base editors tested in this study

**Supplemental Sequence 1. Sequence information of the key elements of base editors tested in this study.**

## TadA8e-nCas9

**NLS-TadA8e V106W -32aa linker-nCas9(D10A)-NLS-NLS**

**PKKKRKVSEVEFSHEYWMRHALTLAKRARDEREVPVGAVLVLNNRVIGEGWNRAIGLHDPTAHAEIMALRQGGLVMQNYRLIDATLYVTFEPCVMCAGAMIHSRIGRVVFGWRNSKRGAAGSLMNVLNYPGMNHRVEITEGILADECAALLCDFYRMPRQVFNAQKKAQSSINSGGSSGGSSGSETPGTSESATPESSGGSSGGSDKKYSIGLAIGTNSVGWAVITDEYKVPSKKFKVLGNTDRHSIKKNLIGALLFDSGETAEATRLKRTARRRYTRRKNRICYLQEIFSNEMAKVDDSFFHRLEESFLVEEDKKHERHPIFGNIVDEVAYHEKYPTIYHLRKKLVDSTDKADLRLIYLALAHMIKFRGHFLIEGDLNPDNSDVDKLFIQLVQTYNQLFEENPINASGVDAKAILSARLSKSRRLENLIAQLPGEKKNGLFGNLIALSLGLTPNFKSNFDLAEDAKLQLSKDTYDDDLDNLLAQIGDQYADLFLAAKNLSDAILLSDILRVNTEITKAPLSASMIKRYDEHHQDLTLLKALVRQQLPEKYKEIFFDQSKNGYAGYIDGGASQEEFYKFIKPILEKMDGTEELLVKLNREDLLRKQRTFDNGSIPHQIHLGELHAILRRQEDFYPFLKDNREKIEKILTFRIPYYVGPLARGNSRFAWMTRKSEETITPWNFEEVVDKGASAQSFIERMTNFDKNLPNEKVLPKHSLLYEYFTVYNELTKVKYVTEGMRKPAFLSGEQKKAIVDLLFKTNRKVTVKQLKEDYFKKIECFDSVEISGVEDRFNASLGTYHDLLKIIKDKDFLDNEENEDILEDIVLTLTLFEDREMIEERLKTYAHLFDDKVMKQLKRRRYTGWGRLSRKLINGIRDKQSGKTILDFLKSDGFANRNFMQLIHDDSLTFKEDIQKAQVSGQGDSLHEHIANLAGSPAIKKGILQTVKVVDELVKVMGRHKPENIVIEMARENQTTQKGQKNSRERMKRIEEGIKELGSQILKEHPVENTQLQNEKLYLYYLQNGRDMYVDQELDINRLSDYDVDHIVPQSFLKDDSIDNKVLTRSDKNRGKSDNVPSEEVVKKMKNYWRQLLNAKLITQRKFDNLTKAERGGLSELDKAGFIKRQLVETRQITKHVAQILDSRMNTKYDENDKLIREVKVITLKSKLVSDFRKDFQFYKVREINNYHHAHDAYLNAVVGTALIKKYPKLESEFVYGDYKVYDVRKMIAKSEQEIGKATAKYFFYSNIMNFFKTEITLANGEIRKRPLIETNGETGEIVWDKGRDFATVRKVLSMPQVNIVKKTEVQTGGFSKESILPKRNSDKLIARKKDWDPKKYGGFDSPTVAYSVLVVAKVEKGKSKKLKSVKELLGITIMERSSFEKNPIDFLEAKGYKEVKKDLIIKLPKYSLFELENGRKRMLASAGELQKGNELALPSKYVNFLYLASHYEKLKGSPEDNEQKQLFVEQHKHYLDEIIEQISEFSKRVILADANLDKVLSAYNKHRDKPIREQAENIIHLFTLTNLGAPAAFKYFDTTIDRKRYTSTKEVLDATLIHQSITGLYETRIDLSQLGGDSGGSKRTADGSEFEPKKKRKV***

## TadA8e-dCpf1

**NLS-TadA8e V106W -32aa linker-dCpf1(D832A/E925A/D1148A) -NLS**

**PKKKRKVSEVEFSHEYWMRHALTLAKRARDEREVPVGAVLVLNNRVIGEGWNRAIGLHDPTAHAEIMALRQGGLVMQNYRLIDATLYVTFEPCVMCAGAMIHSRIGRVVFGWRNSKRGAAGSLMNVLNYPGMNHRVEITEGILADECAALLCDFYRMPRQVFNAQKKAQSSINSGGSSGGSSGSETPGTSESATPESSGGSSGGSSKLEKFTNCYSLSKTLRFKAIPVGKTQENIDNKRLLVEDEKRAEDYKGVKKLLDRYYLSFINDVLHSIKLKNLNNYISLFRKKTRTEKENKELENLEINLRKEIAKAFKGNEGYKSLFKKDIIETILPEFLDDKDEIALVNSFNGFTTAFTGFFDNRENMFSEEAKSTSIAFRCINENLTRYISNMDIFEKVDAIFDKHEVQEIKEKILNSDYDVEDFFEGEFFNFVLTQEGIDVYNAIIGGFVTESGEKIKGLNEYINLYNQKTKQKLPKFKPLYKQVLSDRESLSFYGEGYTSDEEVLEVFRNTLNKNSEIFSSIKKLEKLFKNFDEYSSAGIFVKNGPAISTISKDIFGEWNVIRDKWNAEYDDIHLKKKAVVTEKYEDDRRKSFKKIGSFSLEQLQEYADADLSVVEKLKEIIIQKVDEIYKVYGSSEKLFDADFVLEKSLKKNDAVVAIMKDLLDSVKSFENYIKAFFGEGKETNRDESFYGDFVLAYDILLKVDHIYDAIRNYVTQKPYSKDKFKLYFQNPQFMGGWDKDKETDYRATILRYGSKYYLAIMDKKYAKCLQKIDKDDVNGNYEKINYKLLPGPNKMLPKVFFSKKWMAYYNPSEDIQKIYKNGTFKKGDMFNLNDCHKLIDFFKDSISRYPKWSNAYDFNFSETEKYKDIAGFYREVEEQGYKVSFESASKKEVDKLVEEGKLYMFQIYNKDFSDKSHGTPNLHTMYFKLLFDENNHGQIRLSGGAELFMRRASLKKEELVVHPANSPIANKNPDNPKKTTTLSYDVYKDKRFSEDQYELHIPIAINKCPKNIFKINTEVRVLLKHDDNPYVIGIARGERNLLYIVVVDGKGNIVEQYSLNEIINNFNGIRIKTDYHSLLDKKEKERFEARQNWTSIENIKELKAGYISQVVHKICELVEKYDAVIALADLNSGFKNSRVKVEKQVYQKFEKMLIDKLNYMVDKKSNPCATGGALKGYQITNKFESFKSMSTQNGFIFYIPAWLTSKIDPSTGFVNLLKTKYTSIADSKKFISSFDRIMYVPEEDLFEFALDYKNFSRTDADYIKKWKLYSYGNRIRIFRNPKKNNVFDWEEVCLTSAYKELFNKYGINYQQGDIRALLCEQSDKAFYSSFMALMSLMLQMRNSITGRTDVAFLISPVKNSDGIFYDSRNYEAQENAILPKNADANGAYNIARKVLWAIGQFKKAEDEKLDKVKIAISNKEWLEYAQTSVKHGSPKKKRKV***
