# Supplementary material for: Precise fine-turning of GhTFL1 by base editing tools defines ideal cotton plant architecture
Source: Genome Biol. 2024 Feb 26;25:59. doi: 10.1186/s13059-024-03189-8 (PMC10895741; doi:10.1186/s13059-024-03189-8)
Supplement: Supplementary file 4 — Additional file 4. Review history. [file 13059_2024_3189_MOESM4_ESM.docx]

Review History

**First round of review**

**Reviewer 1**

**Are you able to assess all statistics in the manuscript, including the appropriateness of statistical tests used?** Yes, and I have assessed the statistics in my report.

**Comments to author:**

The manuscript upgraded the adenine base editing system in cotton, summarized the editing efficiency, editing window, and specificity of ABE8, and attempted to expand the PAM range of the system using Cpf1. The author employed this editing tool to carry out small-scale in-situ directed evolution of the promoter and coding regions of the TFL1 gene in cotton, obtaining a variety of mutants, among which L86P and K53G+S78G mutant plants exhibited more desirable plant traits. The molecular mechanisms were also analyzed. This study provides additional evidence for the use of base editing systems to screen for superior alleles of genes through directed evolution, especially for mutations of non-herbicide functional genes. In general, although the manuscript does not show obvious innovations in the development of ABE system, the result that the authors can applied the editor to direct evolution of endogenous genes in cotton is still worthy of affirmation. But the quality of the data and figures should be significantly improved. Together, I think this manuscript could be considered for publication in Genome Biology with appropriate and necessary revisions. Below, please find my specific comments that should be addressed. They are in order line by line, not by importance.

1. In the introduction, some of the references at certain positions may not be suitable.

Yu et al., 2021 and Soyk et al., 2017 may not be suitable. The use of some review-type references may be more appropriate.

When mentioning "CRISPR/Cas9 genome editing is widely used in many crop species to cleave target DNA sites, causing double-strand breaks (DSB) and creating mutants with protein function loss", relevant references should be cited.

(Richter et al., 2020) should be cited after the word "system".

2. "Commonly used base editors, such as the adenine base editor (ABE) and cytosine base editor (CBE), can achieve precise base mutations of C-to-T and A-to-G without inducing DSB", the term "cytidine base editor" is better changed to "cytosine base editor" and placed before ABE to correspond to "enabling precise base mutations of C-to-T and A-to-G without inducing DSB". Additionally, this sentence requires citing relevant references.

3. "These results indicate that the editing activity of GhABE8e is not affected by targeting multiple sites simultaneously, nor does it have a preference for the location of sgRNA.", the authors only tested a set of multiple target sites without altering their position. Therefore, to reach such a conclusion, this set of target sites needs to be transferred to different locations for serial testing.

4. "The sgRNA8 target site has an average editing efficiency of 52.28% and a sgRNA10 target site with 93.91% efficiency," where "sgRNA 10" should be "sgRNA 11".

5. "One sgRNA with TTTV-PAMs (sgRNA12) targeting..." should be changed to "One crRNA with TTTV-PAMs targeting...".

6. "GhABE8e-dCpf1 exhibited editing efficiency of 1.5% at sgRNA12 target site, which is approximately 5-fold higher than the GhABE7.10dCpf1 system (0.2-0.5%)…" Does the author compare these two editors side by side? If not, I think it is imprudent to draw such a conclusion. While ABE8 has been shown to perform better than ABE7.10 in other species, it is not reasonable to directly compare the efficiency between two experiments that were conducted in different years.

7. How did the author construct two or three targets? What is the randomness and coverage? What is the specific target information of the 26 vectors? More information should add in the methods and Supplementary.

8. Did the author transform the sense target and the antisense target together? The theory is that there will be a deletion event, has the author detected such a mutation in the plants? What is the efficiency?

9. "what's more, the editing efficiency of these two loci is 21% and 99%" should be "What's more…".

10. How do the authors identify T0 generation regeneration plants? Have all T0 generation plants undergone genotypic identification? Or do they observe the phenotype first, and then perform genotypic identification on these plants? In any case, I think the authors should use a diagram to clearly list the mutations of the tested plants, including the location of the mutations, the frequency of mutations, whether it is heterozygous or homozygous (I did not see a description of the genotype in the article, but it is actually very important for plant genome editing), etc., so that readers can understand intuitively. I think the T0 data would be better represented as a scattered bar plot, with mutation frequency on the y-axis and individual plants as dots. Then the readers can appreciate the level of mutagenesis and the genotype in the plants.

11. How do the knockout mutation and K53G+S78G mutation interact with GhAP1 and 14-3-3? reduced? or abolished?

12. The authors only made a prediction based on the structure L86, and whether there was any effect or change in structure after the mutation of L86 to P.

13. There are some vectors names and sequences such as GhACBE, GhCABE and GhA3A*ABE in the Supplementary sequences, tut theseinformation have nothing to do with the research content of the paper, please check.

14. In the final model figure, AP1, SOC1 and LFY appeared as genes related to flower development that may be regulated by GhTFL1. So, in the GhTFL1 mutants created by base editing, what is the up-regulated or down-regulated ratio of the relative expression of these genes compared with that of wild type Jin668?

15. The following comments are about figures:

a) Figure 1a, "7.10TadA" can be changed to "TadA7.10", "8eTadA V106W" can be changed to "TadA8e V106W";

b) Figure 1b, the vector name should be added (also in figure 2e), which also should be consistent with the supplementary sequences; unify the spacing of each component;

c) Figure 1c, "GhABE7.10n" should be "GhABE7.10";

d) Figure 2a, 2e, unify the spacing of each component;

e) Figure 2b, "14.4-80.6" should be "14.4-80.6%";

f) Figure 2c, 2f, the size of symbol point is not consistent;

g) Figure 2g, horizontal coordinate words can be arranged diagonally under the coordinate, rather than some above the coordinate;

h) Figure 4a, "promotor" should be "promoter"; the specific sgRNA name can be added on the target diagram, such as sgRNA1, this will allow for a clearer comparison with Figure 4b;

i) Figure 4b, what is the meaning of the different color point, one point stands for one plant?

j) Figure 5b, what is meaning of "GhTFL1L86P-1,-2,3", the different T0 line? Apart from T to C, there is "A" to "G" mutation in the last reads of the GhTFL1L86P-2, how to explain?

k) Figure 5e, "GhTFL1pro783" or "GhTFL1pro-783";

l) Figure 6c, "plant height" should be "Plant height (cm)";

m) Figure 6e, what does the ordinate represent?

n) Supplementary Figure 4, the figure legend "Data are collected from one biological replicate in Figure 3b." should be "Data are collected from one biological replicate in Figure 2f."; the "sgRNA" in the figure should be "crRNA";

o) Supplemental Figure S6, the title "knockdown" should be changed to "knockout"; each small figure should be marked with the corresponding a,b,c,d… and the corresponding figure legends should be added.

p) Supplemental Figure S9, could add more information and description.

I think there is no need 8 figures in main text, 5 or 6 may be more suitable. Such as figure 1 and figure 2 can be combined; 2e and 2f can be moved to Supplementary; figure 8 can be moved as the last term of figure 7.

**Reviewer 2**

**Are you able to assess all statistics in the manuscript, including the appropriateness of statistical tests used?** Yes, and I have assessed the statistics in my report.

**Comments to author:**

This manuscript presents a comprehensive and scientific sound study with a well-designed methodology. The authors successfully optimized the GhABE8 system from Cas9 and Cas12a in cotton to enhance its efficiency. To address the issue of knockout plants with extreme mutant phenotypes, the author devised a multiplex sgRNA strategy targeting the GhTFL1 gene coding sequence as well as the upstream 1000bp promoter region. Through this approach, the GhTFL1 gene were fine-tuned, resulting in new cotton germplasm with moderate height and compact architecture. Moreover, the underlined molecular mechanism was revealed, which provided insights into the nutritional growth pathway in cotton. In summary, the manuscript demonstrated an excellent example of base editors-mediated rapid evolution of agronomical important traits in crops, even in the species traditionally hard to be manipulated with genetic engineering way. Although the science presented is indeed intriguing, the written should be improved before publication.

1. In the results, some descriptions of methodology, such as lines 54 to 58 in the first paragraph, should move to materials and methods part.

2. In the "TadA8e is compatible with dCpf1 protein for efficient base editing" paragraph, there were at least three literatures published in early of 2023, which all showed relatively high efficiency of Cas12a-ABE8e. The necessary citations should be added.

3. What is the principle of selection 26 sgRNAs? What is the coverage of the screening. This information should be presented in the manuscript.

4. In figure 2 (?, the number should be indicated in the figures), I am not sure whether the illustration of multiplex sgRNA expression cassette is correct or not. Since polyTs were inserted, the sgRNA4, 6, 7 may not expressed.

5. There are some language and grammar mistakes through the manuscript, such as

Abstract: line 35 "other proteins";

Page 13, line 42 and line 46, too many "among them" and the line 54 to 59 is confusion.

Page 14, line 10, "those plants were edited"; line 52 to line 56 should rewritten.

**Reviewer 3**

**Are you able to assess all statistics in the manuscript, including the appropriateness of statistical tests used?** Yes, and I have assessed the statistics in my report.

**Comments to author:**

This study aims to devise a proficient tool for cotton base editing, facilitating the targeted modification of endogenous genes to generate mutants with desirable traits. The manuscript is well-structured and thorough, delineating potential applications in cotton base editing tool development and mutant creation. The authors have crafted a series of cotton-specific base editing tools, evaluating their efficacy at endogenous gene loci. Results indicate that GhABE8e displays superior A-to-G editing efficiency with reduced off-target risks. GhABE8e was then employed for saturation mutagenesis in the promoter and coding sequence of the GhTFL1 gene, yielding mutants with favorable agronomic traits. Additionally, an in-depth analysis of the GhTFL1 weak-function mutant GhTFL1L86P was conducted to elucidate its molecular mechanisms. In conclusion, this manuscript provides valuable insights into the development of an efficient cotton base editing tool and its role in mutant creation. Therefore, I recommend that the manuscript be considered for publication in Genome Biology.

To enhance the clarity and academic quality of the paper, I suggest the following revisions:

1. Figure 2 lacks essential details, specifically for sgRNA8, sgRNA9, sgRNA10, and sgRNA11, which appear in Fig. 2c without corresponding schematic representations in Fig. 2a. This information should be included for clarity.

2. In Fig. 2f, GhABE8e-dCpf1 exhibits relatively low editing efficiency at sgRNA12. However, assessing the activity solely based on a single locus is less comprehensive. I suggest that the authors could provide a little more data from various loci to mitigate locus-specific effects when evaluating the editing efficiency of GhABE8e-dCpf1 in cotton.

3. Figure 4 indicates that GhABE8e has extremely low off-target rates at both DNA and RNA levels, which contrasts with similar conclusions in rice. This discrepancy should be discussed, considering relevant ABE8e studies in other species (Li et al., Genome Biol 23, 51 (2022); Wu et al., Plant Biotechnol J 20, 1670-1682 (2022)).

4. For the mutants sg-1 and sg-4-5, which show editing in their GhTFL1 promoter regions, the manuscript should provide gene expression data of GhTFL1 to assess the phenotype variation attributed to upregulation or downregulation of GhTFL1 expression. Moreover, discuss current studies on promoter editing, as referenced in the manuscript (Song et al., Nat Biotechnol 40, 1403-1411 (2022); Zhou et al., Nat Plants 9, 588-604 (2023)).

5. In Fig. 7, several instances do not adhere to scientific standards. The author should make revisions to improve the manuscript's rigor. For instance, in Fig. 7a and Fig. 7b, the final images lack scale bars; the author should incorporate appropriate scale bars within the images and remove the hands appearing at the bottom of the images. In Fig. 7d, each biological replicate for the samples appears different. The author should annotate the 'n' value for each sample at suitable positions on the bar graph. The presentation of Figure 7e lacks clarity and does not effectively convey information to readers. The authors should reconfigure the figure to make it more informative and reader-friendly, with clear explanations of the sequences on the vertical axis and the numbers on the horizontal axis.

**Authors Response**

**Point-by-point responses to the reviewers’ comments:**

**Reviewer #1:**
The manuscript upgraded the adenine base editing system in cotton, summarized the editing efficiency, editing window, and specificity of ABE8, and attempted to expand the PAM range of the system using Cpf1. The author employed this editing tool to carry out small-scale in-situ directed evolution of the promoter and coding regions of the TFL1 gene in cotton, obtaining a variety of mutants, among which L86P and K53G+S78G mutant plants exhibited more desirable plant traits. The molecular mechanisms were also analyzed. This study provides additional evidence for the use of base editing systems to screen for superior alleles of genes through directed evolution, especially for mutations of non-herbicide functional genes. In general, although the manuscript does not show obvious innovations in the development of ABE system, the result that the authors can applied the editor to direct evolution of endogenous genes in cotton is still worthy of affirmation. But the quality of the data and figures should be significantly improved. Together, I think this manuscript could be considered for publication in Genome Biology with appropriate and necessary revisions. Below, please find my specific comments that should be addressed. They are in order line by line, not by importance.
**Question:** 1. In the introduction, some of the references at certain positions may not be suitable. Yu et al., 2021 and Soyk et al., 2017 may not be suitable. The use of some review-type references may be more appropriate. When mentioning "CRISPR/Cas9 genome editing is widely used in many crop species to cleave target DNA sites, causing double-strand breaks (DSB) and creating mutants with protein function loss", relevant references should be cited. (Richter et al., 2020) should be cited after the word "system".

**Response:** We sincerely appreciate the valuable comments. We have checked the literature carefully and would replace some references and modify the positions of others in the INTRODUCTION section in the revised manuscript. We have added the reference (Wang, J.Y., and Doudna, J.A. (2023)) after the sentence “CRISPR/Cas9 genome editing is widely used in many crop species to cleave target DNA sites, causing double-strand breaks (DSB) and creating mutants with protein function loss”. We have modified the sentence “TadA was further evolved to ABE8e using phage-assisted non-continuous and continuous evolution (PANCE and PACE) (Richter et al., 2020) system” to “TadA was further evolved to ABE8e using phage-assisted non-continuous and continuous evolution (PANCE and PACE) system (Richter et al., 2020)” We also highlighted them with the red front in this revised manuscript.

**Question:** 2. "Commonly used base editors, such as the adenine base editor (ABE) and cytosine base editor (CBE), can achieve precise base mutations of C-to-T and A-to-G without inducing DSB", the term "cytidine base editor" is better changed to "cytosine base editor" and placed before ABE to correspond to "enabling precise base mutations of C-to-T and A-to-G without inducing DSB". Additionally, this sentence requires citing relevant references.

**Response:** Thanks. We totally agree with this important point. We have changed the term "cytidine base editor" with "cytosine base editor" and placed before ABE. Additionally, we have added relevant references followed this sentence in the revised manuscript.

**Question:** 3. "These results indicate that the editing activity of GhABE8e is not affected by targeting multiple sites simultaneously, nor does it have a preference for the location of sgRNA.", the authors only tested a set of multiple target sites without altering their position. Therefore, to reach such a conclusion, this set of target sites needs to be transferred to different locations for serial testing.

**Response:** Thanks for your valuable comments of this point. We think what you said is very correct, so we deleted this conclusion.

**Question:** 4. "The sgRNA8 target site has an average editing efficiency of 52.28% and a sgRNA10 target site with 93.91% efficiency," where "sgRNA 10" should be "sgRNA 11".

**Response:** Thanks. We apologize for this mistake and have corrected the “sgRNA10” into “sgRNA11”.

**Question:** 5. "One sgRNA with TTTV-PAMs (sgRNA12) targeting..." should be changed to "One crRNA with TTTV-PAMs targeting...".

**Response:** Thanks for your valuable comments of this manuscript. According to your suggestion, we have corrected the “One sgRNA with TTTV-PAMs” into “One crRNA with TTTV-PAMs”.

**Question:** 6. "GhABE8e-dCpf1 exhibited editing efficiency of 1.5% at sgRNA12 target site, which is approximately 5-fold higher than the GhABE7.10dCpf1 system (0.2-0.5%)…" Does the author compare these two editors side by side? If not, I think it is imprudent to draw such a conclusion. While ABE8 has been shown to perform better than ABE7.10 in other species, it is not reasonable to directly compare the efficiency between two experiments that were conducted in different years.

**Response:** Thank you for your insightful comments. Upon reflection, we agree that it was imprudent to directly compare the editing efficiencies of GhABE8e-dCpf1 and GhABE7.10dCpf1 without conducting side-by-side experiments under identical conditions. We acknowledge that the comparison made between experiments conducted in different years may not accurately reflect the true performance of these editors in cotton. So, we deleted this conclusion.

**Question:** 7. How did the author construct two or three targets? What is the randomness and coverage? What is the specific target information of the 26 vectors? More information should add in the methods and Supplementary.

**Response:** Thanks. We apologize for not giving these details. Firstly, we utilized web application BEsgRNADe to screen for targets that meet the conditions for ABE8e. Concurrently, we predicted and analyzed important domains in TFL1 CDS (using Phyre; http://www.sbg.bio.ic.ac.uk/phyre2) (Figure R1) and key elements of the TFL1 promoter (using PlantCARE) (Figure R2). Taking into account the results from both analyses, we ultimately selected 26 sgRNAs, of which 9 targeted the promoter region and 17 targeted the CDS region (Table R1). Therefore, the selection of sgRNAs was not entirely random. The 17 sgRNAs on the CDS region cover most of the domains.

In the promoter region, among the 9 sgRNAs (sg-1 to sg-9), sg-1 was constructed into the ABE8e vector as a single-target structure, while sg-2 and sg-3, sg-4 and sg-5, sg-6 and sg-7, sg-8 and sg-9 were constructed into the GhABE8e vector as double targets. In the CDS region, out of the 17 sgRNAs (sg-10 to sg-26), sg-16 and sg-20 were combined as a double target, and sg-23, sg-24, sg-25 were combined as a set of three targets in one GhABE8e vector, with the rest constructed as single targets (Table R1).

In total, 26 sgRNAs were designed for both the promoter and CDS sequences, resulting in 19 vectors after combination. The information for each vector is detailed in the table provided. We will ensure to add more detailed information in the methods section and the supplementary materials (Supplementary Table S8) to provide a comprehensive understanding of the construction of the targets.

Figure R1. The CDS DNA Sequence and sgRNA-Targeting Sites with PAM of GhTFL1 in cotton. TFL1 protein structural domain analysis using phyre2 and targets distribution;

Figure R2. The Promoter and genomic DNA Sequence and sgRNA-Targeting Sites with PAM of GhTFL1 in cotton. (a) Motifs analysis of the GhTFL1 promoter using PlantCARE and targets distribution;

Table R1. The 26 targets information of the 19 vectors.

**Question:** 8. Did the author transform the sense target and the antisense target together? The theory is that there will be a deletion event, has the author detected such a mutation in the plants? What is the efficiency?

**Response:** We thank the reviewer for this useful comment. Yes, we did co-transform the sense and antisense targets together (such as sg-4+sg-5) (Table R1), with the theoretical possibility of observing a deletion event between the targets. However, our analysis of the editing results did not find any such deletion events (Figure R3). After careful consideration and discussion, we believe that this may be due to the fact that cotton is an allopolyploid species with A and D subgenomes. The probability of nCas9 simultaneously targeting two targets within the same subgenome (either A or D) is reduced, which in turn decreases the likelihood of observing deletion events. Therefore, we did not detect any fragment deletions in our results.

Figure R3. Editing outcomes of sg-4 and sg-5 on the genome. sg-4 is an antisense target and sg-5 is a sense target; numbers represent editing efficiency (%).

**Question:** 9. "what's more, the editing efficiency of these two loci is 21% and 99%" should be "What's more…".

**Response:** Thank you for pointing out the typographical error regarding the capitalization in our manuscript. We appreciate your attention to detail and will correct this in the text to read “What’s more…” to ensure it adheres to proper grammatical standards.
**Question:** 10. How do the authors identify T0 generation regeneration plants? Have all T0 generation plants undergone genotypic identification? Or do they observe the phenotype first, and then perform genotypic identification on these plants? In any case, I think the authors should use a diagram to clearly list the mutations of the tested plants, including the location of the mutations, the frequency of mutations, whether it is heterozygous or homozygous (I did not see a description of the genotype in the article, but it is actually very important for plant genome editing), etc., so that readers can understand intuitively. I think the T0 data would be better represented as a scattered bar plot, with mutation frequency on the y-axis and individual plants as dots. Then the readers can appreciate the level of mutagenesis and the genotype in the plants.

**Response:** Thank you for your valuable feedback. During the tissue culture stages, we selected a subset of transformed seedlings to test for editing efficiency, with the preliminary aim of exploring the feasibility of using GhABE8e to induce high-density mutations in GhTFL1. The editing results are presented in Figure R4.

Figure R4. Statistics of editing efficiency for artificial evolution of the CDS and promoter of GhTFL1 via GhABE8e.

We did not perform genotypic identification on all T0 generation plants. Instead, we first observed the phenotype and then carried out genotypic identification on those plants exhibited phenotypic changes. From the deep sequencing results, as shown in Figure R5, the *GhTFL1*^L86P^-1 plant exhibited T-to-C base editing at positions 352 and 353 bp in the CDS region. The editing efficiency at the 352 bp site was 91.55%, and at the 353 bp site, it was 94.69%. Since this plant contains both CC and TC genotypes, it is identified as a chimeric.

Figure R5. The T-to-C base editing led to amino acid substitution from leucine to proline at amino acids position 86 of GhTFL1 and the editing efficiency is analyzed by CRISPResso2 for quantification.

We acknowledge the importance of providing a clear description of the genotype for plant genome editing studies, as you have pointed out. To address this, we made a table to display the data with details on the mutation locations, mutation frequencies, and whether the mutations are heterozygous or homozygous for the corresponding individual plants. The results show that the editing patterns observed in the T0 generations tested all exhibit chimerism. We also add additional information (Supplementary Table S3) to this revised manuscript and highlighted them with the red front.

| GhABE8e-mediated base editing in GhTFL1 T0 lines | | | | |
| --- | --- | --- | --- | --- |
| Examined plants | Target | Mutant | | |
|  |  | Editing efficiency | He | Ch |
| GhTFL1^L86P^-1 | sg-17 | 94.69% |  | √ |
| GhTFL1^L86P^-2 | sg-17 | 94.81% |  | √ |
| GhTFL1^L86P^-3 | sg-17 | 93.38% |  | √ |
| GhTFL1^K53G+S78G^-1 | sg-16 | 21.53% |  | √ |
| GhTFL1^K53G+S78G^-1 | sg-20 | 93.32% |  | √ |
| GhTFL1^pro783^_T0-1 | sg-1 | 87.51% |  | √ |
| GhTFL1^pro783^_T0-2 | sg-1 | 85.74% |  | √ |
| GhTFL1^pro485-504^_T0-1 | sg-4 | 75.55% |  | √ |
| GhTFL1^pro485-504^_T0-1 | sg-5 | 94.27% |  | √ |

**Question:** 11. How do the knockout mutation and K53G+S78G mutation interact with GhAP1 and 14-3-3? reduced? or abolished?

**Response:** Thank you for your inquiry regarding the interaction between the K53G+S78G mutation and the GhAP1 and 14-3-3 proteins. We have supplemented our study with additional data on this interaction. The results from our bimolecular fluorescence complementation (BiFC) and luciferase complementation imaging (LCI) assays indicate that the K53G+S78G mutation does not affect the interaction with GhAP1 and 14-3-3, nor the location of the interaction (Figure R6).

Figure R6. BiFC assay A firefly LCI assay confirms that GhTFL1^K53G+S78G^ interact with GhAP1 and Gh14-3-3.

**Question:** 12. The authors only made a prediction based on the structure L86, and whether there was any effect or change in structure after the mutation of L86 to P.

**Response:** Thank you for your insightful query regarding the structural impact of the L86 to P mutation in TFL1. We have utilized AlphaFold (<https://colab.research.google.com/github/sokrypton/ColabFold/blob/main/AlphaFold2.ipynb>) to predict the structural changes resulting from this mutation and have included these findings in the revised manuscript. Our analysis revealed that the branched-chain amino acid leucine (L86) forms two hydrogen bonds with R140. After the mutation to the cyclic amino acid proline (P86), only one hydrogen bond is maintained with R140. This alteration in hydrogen bonding may contribute to the observed changes in the TFL1 structure (Figure R7).

Figure R7. the structural changes resulting from the L86 to P mutation by AlphaFold.

**Question:** 13. There are some vectors names and sequences such as GhACBE, GhCABE and GhA3A*ABE in the Supplementary sequences, but this information have nothing to do with the research content of the paper, please check.

**Response:** Thank you for bringing this to our attention. We apologize for the oversight and appreciate your diligence in reviewing the supplementary sequences. We will carefully review the supplementary material to ensure that all vector names and sequences included are relevant to the research content of the paper.
**Question:** 14. In the final model figure, AP1, SOC1 and LFY appeared as genes related to flower development that may be regulated by GhTFL1. So, in the GhTFL1 mutants created by base editing, what is the up-regulated or down-regulated ratio of the relative expression of these genes compared with that of wild type Jin668?

**Response:** Thank you for your question regarding the expression levels of *AP1*, *SOC1* and *LFY* in the GhTFL1^L86P^ mutants created by base editing compared to the wild type Jin668. We have quantified the expression of *AP1* (Ghir_A13G007920), *SOC1* (Ghir_A11G009010/Ghir_A11G000880/Ghir_D11G008980/ Ghir_D12G011260) and *LFY* (Ghir_A07G005240/Ghir_D07G005260) in both the GhTFL1^L86P^ and Jin668. Our observations indicate a significant upregulation of the *AP1* gene (Figure R8), which can be attributed to the direct interaction between TFL1 and AP1. For *SOC1*, three genes showed upregulation while one did not exhibit any change in expression (Figure R8). This variation in *SOC1* gene expression may be due to functional divergence that occurred during evolution. *LFY*, being a key transcription factor for floral organ development that does not directly interact with TFL1, did not show significant changes in expression in the shoot apex tissues of the GhTFL1^L86P^ mutants compared to the wild type Jin668 (Figure R8).

Figure R8. The expression (FPKM) of *AP1*, *SOC1* and *LFY* in both the GhTFL1^L86P^ and Jin668.

**Question:** 15. The following comments are about figures:
a) Figure 1a, "7.10TadA" can be changed to "TadA7.10", "8eTadA V106W" can be changed to "TadA8e V106W";

**Response:** Thank you for your suggestion regarding the labeling in Figure 1a. We have made the recommended changes, updating “7.10TadA” to “TadA7.10” and “8eTadA V106W” to “TadA8e V106W” for clarity and consistency.
**Question:** b) Figure 1b, the vector name should be added (also in figure 2e), which also should be consistent with the supplementary sequences; unify the spacing of each component;

**Response:** Thank you for your careful review and valuable feedback regarding Figure 1b and Figure 2e (Supplementary Figure S4 in the revised manuscript and figure). We have added the vector names to both figures to ensure consistency with the supplementary sequences. Additionally, we have adjusted the spacing of each component within the figures for uniformity and better visual presentation.
**Question:** c) Figure 1c, "GhABE7.10n" should be "GhABE7.10";

**Response:** Thank you for your attention to detail. We have made the necessary correction, and “GhABE7.10n” has been updated to “GhABE7.10” in Figure 1c.
**Question:** d) Figure 2a, 2e, unify the spacing of each component;

**Response:** Thank you for your feedback. We have taken note of your suggestion and have ensured that the spacing of each component in Figure 2a and 2e (Figure 1d and Supplementary Figure S4 in the revised manuscript and figure) is now consistent for improved visual presentation.
**Question:** e) Figure 2b, "14.4-80.6" should be "14.4-80.6%";

**Response:** Thank you for bringing this to our attention. We have made the necessary adjustment, and “14.4-80.6” has been updated to “14.4-80.6%” in Figure 2b (Figure 1e in the revised manuscript and figure).
**Question:** f) Figure 2c, 2f, the size of symbol point is not consistent;

**Response:** Thank you for your feedback. We have taken note of your observation and have ensured that the size of the symbol points is now consistent in Figure 2c and 2f (Figure 1f and Supplementary Figure S4 in the revised manuscript and figure) for improved clarity and accuracy.
**Question:** g) Figure 2g, horizontal coordinate words can be arranged diagonally under the coordinate, rather than some above the coordinate;

**Response:** Thank you for your careful review and valuable suggestion regarding the arrangement of the horizontal coordinate labels in our figures. I would like to clarify that there seems to be a misunderstanding as our manuscript does not contain a Figure 2g. Based on the description of the issue you provided, it appears you are referring to Figure 3g. Following your recommendation, we have made the necessary adjustments to Figure 3g (Figure 2g in the revised manuscript and figure), ensuring that the horizontal coordinate words are now arranged diagonally under the coordinate.
**Question:** h) Figure 4a, "promotor" should be "promoter"; the specific sgRNA name can be added on the target diagram, such as sgRNA1, this will allow for a clearer comparison with Figure 4b;

**Response:** Thank you for your valuable feedback. We have made the necessary correction, updating “promotor” to “promoter” in Figure 4a (Figure 3a in the revised manuscript and figure). Additionally, we have added specific sgRNA names to the target diagram to allow for a clearer comparison with Figure 4b (Figure 3b in the revised manuscript and figure).
**Question:** i) Figure 4b, what is the meaning of the different color point, one point stands for one plant?

**Response:** Thank you for your inquiry, and we appreciate the opportunity to clarify this aspect of the figure. The different color points in Figure 4b (Figure 3b in the revised manuscript and figure) represent different cotton plants infected with different vectors. Each point on the graph corresponds to an individual plant.
**Question:** j) Figure 5b, what is meaning of "GhTFL1L86P-1,-2,3", the different T0 line? Apart from T to C, there is "A" to "G" mutation in the last reads of the GhTFL1L86P-2, how to explain?

**Response:** We appreciate your attention to this detail and hope that this additional information addresses your concern. The designations GhTFL1L86P-1, -2, and -3 in Figure 5b (Figure 4b in the revised manuscript and figure) refer to three distinct T0 transgenic lines that were generated in our study. In the case of the GhTFL1L86P-2 line, in addition to the T to C mutation, we observed an A to G mutation present in 36 reads. Given that this mutation accounts for only 0.25% of the total reads, we consider it to be a sequencing error rather than a true mutation event.
**Question:** k) Figure 5e, "GhTFL1pro783" or "GhTFL1pro-783";

**Response:** Thank you for your feedback. We have taken your suggestion into consideration and have decided to maintain “GhTFL1^pro783^” in Figure 5e (Figure 4e in the revised manuscript and figure) consistently throughout both the manuscript and the figures.
**Question:** l) Figure 6c, "plant height" should be "Plant height (cm)";

**Response:** Thank you for your suggestion. We have updated the label to "Plant height (cm)" in Figure 6c (Figure 5c in the revised manuscript and figure).
**Question:** m) Figure 6e, what does the ordinate represent?

**Response:** Thanks. We apologize for not giving this detail. The ordinate in the Figure 6e (Figure 5e in the revised manuscript and figure) represents the target site information, including the 20 bp sgRNA sequence and the adjacent 3 bp PAM sequence, for the T1 generation individual plants of GhTFL1^L86P^ and GhTFL1^K53G+S78G^.
**Question:** n) Supplementary Figure 4, the figure legend "Data are collected from one biological replicate in Figure 3b." should be "Data are collected from one biological replicate in Figure 2f."; the "sgRNA" in the figure should be "crRNA";

**Response:** Thank you for your meticulous attention to the details in our manuscript. We apologize for the oversight. In accordance with the adjustments made to the figures, we have merged Figure 2f with Supplementary Figure 4 to become Supplementary Figure 4. Consequently, we will revise the figure legend to state “Data are collected from one biological replicate in Supplementary Figure 4b.” Additionally, we have corrected the terminology within the figure from “sgRNA” to “crRNA” as you suggested.
**Question:** o) Supplemental Figure S6, the title "knockdown" should be changed to "knockout"; each small figure should be marked with the corresponding a,b,c,d… and the corresponding figure legends should be added.

**Response:** Thank you for your attention to detail in Supplemental Figure S6. We will amend the title from “knockdown” to “knockout” to accurately reflect the nature of the genetic modification. Additionally, we will label each panel with the corresponding a,b,c,d… and provide the necessary figure legends to describe each image clearly. The revised figure legend will read as follows: “**Supplemental Figure S6. Creating extreme dwarf and early flowering cotton using CRISPR/Cas9 knockout of GhTFL1.** (a and b) the wild-type at different periods; (c-g) The knockout line. *GhTFL1-KO* showed an extremely dwarf phenotype, with the apical leaf of the main stem being replaced by a flower bud as an apical flower, while the axillary flower grew directly under the axillary part of the main stem leaf. Scale bars, 10 cm.”.
**Question:** p) Supplemental Figure S9, could add more information and description.

**Response:** Thank you for your suggestion to provide more detailed information and descriptions for Supplemental Figure S9 (Supplementary Figure S10 in the revised manuscript and figure). We have labeled each image with the corresponding lowercase letters (a, b, c, d, ...), and have expanded the figure legends to include comprehensive descriptions of the phenotypes observed.

The revised figure legends will include the following details:

**Supplemental Figure S10**. **Different cotton architecture obtained from directed evolution *GhTFL1* by GhABE8e.** (a-f) The phenotypes resulting from the mutation of the 163rd amino acid F to L in GhTFL1, showing dwarf plants with clustered flower buds at the shoot apex; (g) The phenotype after the mutation of the 89th amino acid I to M in GhTFL1, with increased plant branching and changes in leaf morphology, including deeper leaf margin indentations; (h-j) The phenotypes of plants with the 143rd amino acid F mutated to L in GhTFL1, where plants exhibit shortened fruiting branches compared to the wild type; (k-m) The phenotypes resulting from point mutations in the GhTFL1 promoter, with plants carrying mutations at positions 783 upstream of the ATG (sg-1) showing darker and thicker leaves; plants with mutations at positions 663 and 618 in the promoter exhibit a significant delay in flowering.
**Question:** I think there is no need 8 figures in main text, 5 or 6 may be more suitable. Such as figure 1 and figure 2 can be combined; 2e and 2f can be moved to Supplementary; figure 8 can be moved as the last term of figure 7.

**Response:** Thank you for your suggestion to reduce the number of figures in the text. We appreciate your suggestion and have merged figures 1 and 2, moved figures 2e and 2f to the supplementary information, and moved figure 8 to the last term of figure 7. The figure legends for both the main and supplementary figures have been updated accordingly in the revised manuscript and figure.

**Reviewer #2**:

This manuscript presents a comprehensive and scientific sound study with a well-designed methodology. The authors successfully optimized the GhABE8 system from Cas9 and Cas12a in cotton to enhance its efficiency. To address the issue of knockout plants with extreme mutant phenotypes, the author devised a multiplex sgRNA strategy targeting the GhTFL1 gene coding sequence as well as the upstream 1000bp promoter region. Through this approach, the GhTFL1 gene were fine-tuned, resulting in new cotton germplasm with moderate height and compact architecture. Moreover, the underlined molecular mechanism was revealed, which provided insights into the nutritional growth pathway in cotton. In summary, the manuscript demonstrated an excellent example of base editors-mediated rapid evolution of agronomical important traits in crops, even in the species traditionally hard to be manipulated with genetic engineering way. Although the science presented is indeed intriguing, the written should be improved before publication.

**Question:** 1. In the results, some descriptions of methodology, such as lines 54 to 58 in the first paragraph, should move to materials and methods section.

**Response:** Thank you for your feedback. We appreciate your suggestion to move some of the methodological descriptions from the Results section to the Materials and Methods section. We will carefully review the manuscript and make the necessary adjustments to ensure that the content is organized properly and presented in the most appropriate sections. We also modified these changes in this revised manuscript and highlighted them with the red front.

**Question:** 2. In the "TadA8e is compatible with dCpf1 protein for efficient base editing" paragraph, there were at least three literatures published in early of 2023, which all showed relatively high efficiency of Cas12a-ABE8e. The necessary citations should be added.

**Response:** Thank you for bringing this to our attention. We appreciate your valuable input regarding the need to include citations for the recent literature that demonstrated the relatively high efficiency of Cas12a-ABE8e. We have checked the literature carefully and added the reference [2-4] after the sentence “TadA8e showed efficient A-to-G base editing activity with dLbCas12a (dLbCpf1) for the first time in animal and plant cells”. We also highlighted them with the red front in this revised manuscript.
**Question:** 3. What is the principle of selection 26 sgRNAs? What is the coverage of the screening. This information should be presented in the manuscript.

**Response:** We apologize for not clearly conveying the selection criteria and principles for the 26 sgRNAs in our manuscript. The sgRNAs were chosen based on their predicted on-target efficiency and minimal off-target effects. Firstly, we utilized web application BEsgRNADe to screen for targets that meet the conditions for ABE8e. Concurrently, we predicted and analyzed important domains in TFL1 CDS (using Phyre) (Figure R9) and key elements of the TFL1 promoter (using PlantCARE) (Figure R10). Taking into account the results from both analyses, we ultimately selected 26 sgRNAs, of which 9 targeted the promoter region and 17 targeted the CDS region. Therefore, the selection of sgRNAs was not entirely random. The 17 sgRNAs on the CDS region cover most of the domains.

In the promoter region, among the 9 sgRNAs (sg-1 to sg-9), sg-1 was constructed into the ABE8e vector as a single-target structure, while sg-2 and sg-3, sg-4 and sg-5, sg-6 and sg-7, sg-8 and sg-9 were constructed into the GhABE8e vector as double targets. In the CDS region, out of the 17 sgRNAs (sg-10 to sg-26), sg-16 and sg-20 were combined as a double target, and sg-23, sg-24, sg-25 were combined as a set of three targets in one GhABE8e vector, with the rest constructed as single targets (Table R2).

In total, 26 sgRNAs were designed for both the promoter and CDS sequences, resulting in 19 vectors after combination. The information for each vector is detailed in the table provided. We will ensure to add more detailed information in the methods section and the supplementary materials (Supplementary Table S8) to provide a comprehensive understanding of the construction of the targets.

We will include a detailed explanation of the sgRNA selection process in the methods section and the supplementary materials to provide readers with a comprehensive understanding of our methodology.

Figure R9. The CDS DNA Sequence and sgRNA-Targeting Sites with PAM of *GhTFL1* in cotton. TFL1 protein structural domain analysis using phyre2 and targets distribution;

Figure R10. The Promoter and genomic DNA Sequence and sgRNA-Targeting Sites with PAM of *GhTFL1* in cotton. (a) Motifs analysis of the *GhTFL1* promoter using PlantCARE and targets distribution;

Table R2. The 26 targets information of the 19 vectors.

**Question:** 4. In figure 2 (?, the number should be indicated in the figures), I am not sure whether the illustration of multiplex sgRNA expression cassette is correct or not. Since polyTs were inserted, the sgRNA4, 6, 7 may not expressed.

**Response:** Thank you for bringing this to our attention. We apologize for the incorrect labeling of polyTs in the vector diagram. We have corrected this error in the revised figure (Figure 1d in the revised manuscript and figure) to accurately represent the multiplex sgRNA expression cassette.
**Question:** 5. There are some language and grammar mistakes through the manuscript, such as Abstract: line 35 "other proteins";

**Response:** Thank you for pointing out the typographical error in the abstract. We have corrected “other protein” to “other proteins” as per your observation. We have highlighted the revised text in red in the manuscript.
**Question:** Page 13, line 42 and line 46, too many "among them" and the line 54 to 59 is confusion.

**Response:** Thank you for your feedback. We have revised the sentence as per your suggestion. The sentence “Among them, some plants exhibiting obvious phenotypes (such as inflorescence structure, plant height, or architecture) were isolated for further analysis (Figure 5 and Supplementary Figure S9). Among them, the plants GhTFL1L86P carrying two non-synonymous mutations (from T to C) at the 352-bp and 353-bp positions, which resulted in an amino acid substitution from L to P at the 86th position, showed determinate main shoots with clustered fruiting bolls and rare twin flowers” has been modified to “Some plants exhibiting obvious phenotypes, such as changes in inflorescence structure, plant height, or architecture, were isolated for further analysis (Figure 4 and Supplementary Figure S10). Specifically, the plants *GhTFL1*^L86P^ carried two non-synonymous mutations (from T to C) at the 352-bp and 353-bp positions, resulting in an amino acid substitution from L to P at the 86th position. These plants showed determinate main shoots with clustered fruiting bolls and rare twin flowers.” We have highlighted the revised text in red in the manuscript.

Thank you for bringing the confusion in lines 54 to 59 to our attention. We have revised the sentence for clarity. The sentence has now been simplified to “The editing efficiency of the plants *GhTFL1*^L86P^ mutation was up to 94.8% (Figure 4a, b and Supplementary Table S3).” We have highlighted this revision in red in the revised manuscript to ensure that the changes are easily identifiable.
**Question:** Page 14, line 10, "those plants were edited"; line 52 to line 56 should rewritten.

**Response:** Thank you for your careful review and valuable comments. We have addressed the grammatical issue you highlighted on page 14, line 10. The original sentence “In plants with high density mutations in the promoter region of the GhTFL1 gene, The plants that were edited at 783 bp upstream of the ATG in GhTFL1 (GhTFL1^pro783^) exhibited a phenotype characterized by smaller leaves with darker colors.” has been revised to “In plants with a high density of mutations in the promoter region of the *GhTFL1* gene, the individual that was edited at 783 bp upstream of the ATG start codon (*GhTFL1*^pro783^_T0-1) exhibited a phenotype characterized by smaller, darker-colored leaves” We have highlighted the revised sentence in red in the revised manuscript to facilitate easy identification of the change.

We have rewritten the sentence to enhance clarity and accurately convey our findings. The updated sentence is as follows:“The T1 generation of *GhTFL1*^K53G+S78G^ exhibits a more pronounced phenotype than the T0 generation, with fruit branches that are shortened and terminate in clusters. Additionally, the apical meristems of the T1 generation of *GhTFL1*^K53G+S78G^ maintain indeterminate growth, resulting in a semi-dwarf stature of the plants when compared to the wild-type Jin668.” We have highlighted this revision in red in the revised manuscript to facilitate easy recognition of the changes.

**Reviewer #3:**

This study aims to devise a proficient tool for cotton base editing, facilitating the targeted modification of endogenous genes to generate mutants with desirable traits. The manuscript is well-structured and thorough, delineating potential applications in cotton base editing tool development and mutant creation. The authors have crafted a series of cotton-specific base editing tools, evaluating their efficacy at endogenous gene loci. Results indicate that GhABE8e displays superior A-to-G editing efficiency with reduced off-target risks. GhABE8e was then employed for saturation mutagenesis in the promoter and coding sequence of the GhTFL1 gene, yielding mutants with favorable agronomic traits. Additionally, an in-depth analysis of the GhTFL1 weak-function mutant GhTFL1L86P was conducted to elucidate its molecular mechanisms. In conclusion, this manuscript provides valuable insights into the development of an efficient cotton base editing tool and its role in mutant creation. Therefore, I recommend that the manuscript be considered for publication in Genome Biology.

To enhance the clarity and academic quality of the paper, I suggest the following revisions:

**Question:** 1. Figure 2 lacks essential details, specifically for sgRNA8, sgRNA9, sgRNA10, and sgRNA11, which appear in Fig. 2c without corresponding schematic representations in Fig. 2a. This information should be included for clarity.

**Response:** Thank you for pointing out the missing details in Figure 2. We have taken your feedback into consideration and have revised the figure accordingly. To improve clarity and coherence, we have merged Figure 2 with Figure 1 and included the corresponding schematic representations for sgRNA8, sgRNA9, sgRNA10, and sgRNA11 in Figure 1d.
**Question:** 2. In Fig. 2f, GhABE8e-dCpf1 exhibits relatively low editing efficiency at sgRNA12. However, assessing the activity solely based on a single locus is less comprehensive. I suggest that the authors could provide a little more data from various loci to mitigate locus-specific effects when evaluating the editing efficiency of GhABE8e-dCpf1 in cotton.

**Response:** Thank you for your insightful comments regarding the evaluation of the editing efficiency of GhABE8e-dCpf1 in cotton as presented in Fig. 2f (Supplementary Figure S4 in the revised manuscript and figure). We understand your concern about the comprehensiveness of assessing the activity based on a single locus. Due to the time constraints associated with cotton tissue culture, we have currently only tested the editing efficiency at the sgRNA12 target site for GhABE8e-dCpf1. We acknowledge the importance of evaluating multiple loci to mitigate locus-specific effects and provide a more thorough assessment. We plan to include additional data from various loci in future studies to comprehensively evaluate the editing efficiency of GhABE8e-dCpf1.
**Question:** 3. Figure 4 indicates that GhABE8e has extremely low off-target rates at both DNA and RNA levels, which contrasts with similar conclusions in rice. This discrepancy should be discussed, considering relevant ABE8e studies in other species (Li et al., Genome Biol 23, 51 (2022); Wu et al., Plant Biotechnol J 20, 1670-1682 (2022)).

**Response:** Thank you for your insightful observations regarding the off-target of GhABE8e as presented in Figure 4 (Figure 2 in the revised manuscript and figure), and for drawing comparisons with similar studies in rice. We recognize the importance of discussing these discrepancies and have included an additional discussion in our manuscript to address this matter.

We have added the sentence

“The adverse effects of off-target mutations on plant functional genomics research and molecular breeding practices based on genome editing strategies cannot be overlooked. Studies in rice have detected A-to-G off-target mutations in single plants edited with SpCas9n-TadA8e, and have found a correlation between the off-target effects of ABE8e and its expression levels[5]. Additionally, using rice as a model for deciphering the off-target effects of plant genome editing mediated by the PAM-relaxed adenine base editor (nSpRY-ABE8e), it was observed that nSpRY-ABE8e-edited rice plants produced ABE8e-dependent off-target mutations at the genome-wide level[6]. In our study, we conducted a comprehensive assessment of the specificity of GhABE8e (V106W) at both DNA and RNA levels in allopolyploid cotton using deeply sequenced genomes and transcriptomes. Our results revealed that GhABE8e did not induce sgRNA-dependent off-target effects at either the DNA or RNA level. Furthermore, the optimized deaminase TadA8e (V106W) used in GhABE8e did not exhibit sgRNA-independent off-target effects at the DNA level. Regarding RNA-level sgRNA-independent off-target effects, our analysis indicated that A-to-G off-target mutations induced by deaminases TadA8e accounted for less than 1% of all single nucleotide variants detected in the transcriptome, which differs from the results observed with ABE8e in rice. The differences in specificity between ABE8e in the allopolyploid cotton genome and the diploid rice genome may be attributed to the complexity of the cotton genome, which contains a large number of repetitive sequences and homologous genes, potentially increasing the number of target sites and enhancing the action of GhABE8e. Additionally, the chromatin structure and epigenetic features of the cotton genome, such as DNA methylation and histone modifications, may influence the specificity of the editing tool. Furthermore, the process of tissue culture may also affect the specificity of genome editing, as tissue culture conditions can induce changes in the physiological state and gene expression patterns of cells, potentially influencing the interaction between the editing tool and its targets. It is important to note that non-specific mutations have relatively limited negative effects in plant breeding, and base mutations at the RNA level are not stably inherited in plants. Therefore, a higher tolerance for off-target effects of ABE8e in plants may be warranted.” into Discussion section in this revised manuscript.

**Question:** 4. For the mutants sg-1 and sg-4-5, which show editing in their GhTFL1 promoter regions, the manuscript should provide gene expression data of GhTFL1 to assess the phenotype variation attributed to upregulation or downregulation of GhTFL1 expression. Moreover, discuss current studies on promoter editing, as referenced in the manuscript (Song et al., Nat Biotechnol 40, 1403-1411 (2022); Zhou et al., Nat Plants 9, 588-604 (2023)).

**Response:** Thank you for your valuable suggestion. We have taken your feedback into consideration and have included the expression data of sg-1 and sg-4-5 in the revised manuscript (Table R11).

We have added the sentence and figure

“During the flowering period, the *GhTFL1*^pro783^_T0-1 exhibited smaller inflorescences, which resulted in a failure to produce fruit. Consequently, we selected three T0 generation plants (*GhTFL1*^pro783^_T0-1, *GhTFL1*^pro783^_T0-2 and *GhTFL1*^pro783^_T0-3) of *GhTFL1*^pro783^ for the assessment of *GhTFL1* gene expression levels. Additionally, we evaluated the expression levels in T0 generation single plants of *GhTFL1*^pro485-504^ and two T1 generation descendants. The results indicated an increase in *GhTFL1* expression in all three T0 generation *GhTFL1*^pro783^ mutants, with *GhTFL1*^pro783^_T0-1 and *GhTFL1*^pro783^_T0-1 showing a significant upregulation of *GhTFL1* expression compared to the Jin668 control. The *GhTFL1* expression in the T0 generation of *GhTFL1*^pro485-504^_T0-1 was significantly higher than that in the wild type, and this upregulation was also observed in the corresponding T1 generation progeny (Supplementary Figure S11)” into Result section in this revised manuscript.

Figure R11. Relative expression of *GhTFL1* at the RNA level in WT, the T0 and the T1 prohenies from the promoter mutant of GhTFL1. *GhUBQ* was used as internal control.

And added the sentence:

“The ability to effectively edit plant gene promoters, thereby altering the expression levels or patterns of target genes, is emerging as a critical tool for elucidating the biological functions of these promoters. Moreover, the creation of promoter-edited germplasm with valuable traits for breeding is becoming an increasingly important area of focus. Recent advancements have demonstrated that targeted editing of gene promoter regions using Cas9 and Cas12a genome editing technologies can enhance or improve crop agronomic traits without adversely affecting other characteristics[7-10]. In light of these developments, our study employed GhABE8e to introduce point mutations into the promoter region of the GhTFL1 gene, leading to observable phenotypic changes. Although the constraints of GhABE8e’s PAM specificity and mutation types limited our ability to generate a broad spectrum of continuous phenotypes, our findings provide preliminary evidence supporting the feasibility of using base editing tools for promoter region modifications. This approach holds promise for refining gene expression in a precise and controlled manner, which is essential for both fundamental research and the development of improved crop varieties.” into Discussion section in this revised manuscript.

**Question:** 5. In Fig. 7, several instances do not adhere to scientific standards. The author should make revisions to improve the manuscript's rigor. For instance, in Fig. 7a and Fig. 7b, the final images lack scale bars; the author should incorporate appropriate scale bars within the images and remove the hands appearing at the bottom of the images. In Fig. 7d, each biological replicate for the samples appears different. The author should annotate the 'n' value for each sample at suitable positions on the bar graph. The presentation of Figure 7e lacks clarity and does not effectively convey information to readers. The authors should reconfigure the figure to make it more informative and reader-friendly, with clear explanations of the sequences on the vertical axis and the numbers on the horizontal axis.
**Response:** Thank you for your critical evaluation of Figure 7 in our manuscript. Upon reviewing your comments, we believe there may have been a reference error, and you are likely addressing concerns related to Figure 6. We take your comments seriously and have made the following revisions to enhance the quality and clarity of the Figure 6 (Figure 5 in the revised manuscript and figure):

In Fig. 5a and Fig. 5b, we have now included appropriate scale bars to provide a clear reference for the size of the visualized features. Additionally, we have removed the hands from the bottom of the images to maintain a professional and focused presentation of the data.

In Fig. 5d, we have annotated the ‘n’ value for each sample to accurately represent the number of biological replicates included in the analysis. This annotation has been placed in a suitable position on the bar graph to ensure that it is easily visible and does not obstruct other important information.

We have reconfigured Fig. 5e to improve its informativeness and readability. The sequences on the vertical axis and the numbers on the horizontal axis have been clearly labeled and explained, ensuring that the figure effectively communicates the intended information to the readers. Amend the figure legends for Figure 5e to read as follows:
**(e)** The genotype analysis of the T1 progenies of *GhTFL1*^L86P^ and *GhTFL1*^K53G+S78G^. The ordinate represents the target site information, including the 20 bp sgRNA sequence and the adjacent 3 bp PAM sequence, for the T1 generation individual plants of *GhTFL1*^L86P^ and *GhTFL1*^K53G+S78G^. Each dot represents the editing efficiency of an independent sample.

**References**

1.	Mirdita M, Schütze K, Moriwaki Y, Heo L, Ovchinnikov S, Steinegger M: **ColabFold: making protein folding accessible to all.** *Nature Methods* 2022, **19:**679-682.

2.	Wang H, Liang J, Chen L, Deng B, Gu D, Liu X, Jin S, Xu R, Qin R, Zhu Y, et al: **Development of plant cytosine base editors with the Cas12a system.** *The Crop Journal* 2023, **11:**1451-1457.

3.	Gaillochet C, Peña Fernández A, Goossens V, D’Halluin K, Drozdzecki A, Shafie M, Van Duyse J, Van Isterdael G, Gonzalez C, Vermeersch M, et al: **Systematic optimization of Cas12a base editors in wheat and maize using the ITER platform.** *Genome Biology* 2023, **24:**6.

4.	Cheng Y, Zhang Y, Li G, Fang H, Sretenovic S, Fan A, Li J, Xu J, Que Q, Qi Y: **CRISPR-Cas12a base editors confer efficient multiplexed genome editing in rice.** *Plant Commun* 2023, **4:**100601.

5.	Li S, Liu L, Sun W, Zhou X, Zhou H: **A large-scale genome and transcriptome sequencing analysis reveals the mutation landscapes induced by high-activity adenine base editors in plants.** *Genome Biology* 2022, **23:**51.

6.	Wu Y, Ren Q, Zhong Z, Liu G, Han Y, Bao Y, Liu L, Xiang S, Liu S, Tang X, et al: **Genome-wide analyses of PAM-relaxed Cas9 genome editors reveal substantial off-target effects by ABE8e in rice.** *Plant Biotechnology Journal* 2022, **20:**1670-1682.

7.	Zhou J, Liu G, Zhao Y, Zhang R, Tang X, Li L, Jia X, Guo Y, Wu Y, Han Y, et al: **An efficient CRISPR–Cas12a promoter editing system for crop improvement.** *Nature Plants* 2023, **9:**588-604.

8.	Song X, Meng X, Guo H, Cheng Q, Jing Y, Chen M, Liu G, Wang B, Wang Y, Li J, Yu H: **Targeting a gene regulatory element enhances rice grain yield by decoupling panicle number and size.** *Nature Biotechnology* 2022, **40:**1403-1411.

9.	Liu L, Gallagher J, Arevalo ED, Chen R, Skopelitis T, Wu Q, Bartlett M, Jackson D: **Enhancing grain-yield-related traits by CRISPR–Cas9 promoter editing of maize CLE genes.** *Nature Plants* 2021, **7:**287-294.

10.	Hendelman A, Zebell S, Rodriguez-Leal D, Dukler N, Robitaille G, Wu X, Kostyun J, Tal L, Wang P, Bartlett ME, et al: **Conserved pleiotropy of an ancient plant homeobox gene uncovered by cis-regulatory dissection.** *Cell* 2021, **184:**1724-1739.e1716.

**Second round of review**

**Reviewer 1**

After reading the related information carefully, the author added clear description on most of my questions and did their best to improve their manuscript. There are still several issues that need to be addressed.

Q1. The mutant genotype, mutation frequency and transmission of the edits in next generation are still unclear.

Of the 200 T0 regenerated plants, how many plant genotypes were identified and what was the specific mutation type of each T0 plant (on which specific sgRNA ? at which specific location of the sgRNA was the “A”mutation)?

What is the final genotype (homozygous, heterozygous, chimeric or wild type)?

What about the transmission? How many T0 plants were identified? How many corresponding T1 are identified for each T0? What are the genotypes, and do they correspond to Mendelian proportions?

Therefore, it is suggested that the author summarize these specific information into the corresponding figure or table, so that the reader can clearly understand.

Q2. How to transfer 26 vectors into plants by Agrobacterium transformation method? Is each vector transformed into Agrobacterium and then mixed in equal proportions before infection? Or will 26 vectors be mixed in equal proportions and then transformed into Agrobacterium to infection? The authors can add more detail information in the Method part.

Q3. In Figure 3b, the author designed a total of 26 sgRNAs, but only presented mutations of 13 sgRNAs. Did the rest have no mutations? If there are other sgRNA mutations, it is recommended to list them all. If not, you can explain it in the main text and suggest that the order of the horizontal coordinate sgRNA is arranged according to the size of the number (sg-1，-2，-3，-4，-5，-11，-15，-16，-17，-19，-20，-21，-22), and the size of the points in the figure is inconsistent, such as purple and green points are sometimes large and sometimes small, please unify.

Q4. In Figure 1e, it is suggested to mark the mutation of A in which positions with red or underline, which is more intuitive.

**Reviewer 3**

Upon evaluating the revised manuscript and the authors' responses to previous comments, I am pleased to see that the authors have addressed most of the concerns raised in the initial review. The revisions made to the manuscript, particularly in areas of experimental design, data presentation, and addressing specific technical questions, have improved the clarity and scientific rigor of the study. These revisions contribute substantially to the manuscript's quality, making it a valuable addition to the field. Given these improvements and the authors' diligent efforts to address the concerns, I believe that the revised manuscript now meets the publication standards for Genome Biology.

**Authors Response**

**Point-by-point responses to the reviewers’ comments:**

**Reviewer #1:** After reading the related information carefully, the author added clear description on most of my questions and did their best to improve their manuscript. There are still several issues that need to be addressed.
Q1. The mutant genotype, mutation frequency and transmission of the edits in next generation are still unclear.
Of the 200 T0 regenerated plants, how many plant genotypes were identified and what was the specific mutation type of each T0 plant (on which specific sgRNA ? at which specific location of the sgRNA was the “A” mutation)?
What is the final genotype (homozygous, heterozygous, chimeric or wild type)?
What about the transmission? How many T0 plants were identified? How many corresponding T1 are identified for each T0? What are the genotypes, and do they correspond to Mendelian proportions?
Therefore, it is suggested that the author summarize these specific information into the corresponding figure or table, so that the reader can clearly understand.
**Response:** We sincerely appreciate the valuable comments. We have screened a total of 244 T0 individual plants, which encompass information for 17 sgRNAs. We have compiled the target information and the final genotypes of these plants into a detailed table for clarity (Additional file 2: Table S3). This table provides specific mutation types for each T0 plant, including the sgRNA involved and the precise location of the “A” mutation within the sgRNA target site, as well as the final genotype (homozygous, heterozygous, or chimeric) of each plant. Regarding the transmission of edits to the T1 generation, we have identified 71 individual T1 progeny corresponding to 7 targeted genes from the T0 plants. We have performed positive identification for these 71 T1 individuals. The results of the positive identification also have been organized into Additional file 2: Table S3.

Q2. How to transfer 26 vectors into plants by Agrobacterium transformation method? Is each vector transformed into Agrobacterium and then mixed in equal proportions before infection? Or will 26 vectors be mixed in equal proportions and then transformed into Agrobacterium to infection? The authors can add more detail information in the Method part.
**Response:** Thanks for your valuable comments of this point. To clarify, we utilized a total of 19 vectors to target the 26 sgRNAs. Each vector was individually transformed into *Agrobacterium tumefaciens*. Following this, we proceeded with the independent infection of cotton hypocotyls using each *Agrobacterium* strain containing the respective vector. We acknowledge the importance of providing detailed methodologies to ensure the reproducibility and clarity of our experimental procedures. In response to your suggestion, we have added this sentence (“The 19 constructed vectors were transferred to *Agrobacterium* strains using the electroporation method. Subsequently, these 19 *Agrobacterium* strains containing the GhABE8e vector were individually used to infect cotton hypocotyls in sequence.”) in the Methods section of our manuscript to include a more comprehensive description of the transformation process, ensuring that each step is clearly articulated.

Q3. In Figure 3b, the author designed a total of 26 sgRNAs, but only presented mutations of 13 sgRNAs. Did the rest have no mutations? If there are other sgRNA mutations, it is recommended to list them all. If not, you can explain it in the main text and suggest that the order of the horizontal coordinate sgRNA is arranged according to the size of the number (sg-1，-2，-3，-4，-5，-11，-15，-16，-17，-19，-20，-21，-22), and the size of the points in the figure is inconsistent, such as purple and green points are sometimes large and sometimes small, please unify.
**Response:** Thanks for your valuable comments of this manuscript. We apologize for any confusion caused by the presentation of the sgRNA mutations in Figure 3b. During the tissue culture phase, we performed mutation screening on the callus tissues and did not detect mutations for four targets: sg-6, sg-7, sg-8, and sg-10. Aside from these four targets, we have reorganized the mutation information for the remaining 22 targets and have now included all of them in Figure 3b. The sgRNAs are arranged in numerical order from sg-1 to sg-26 for clarity. Additionally, we have noticed the inconsistency in the size of the points representing different sgRNA mutations in the figure, as you pointed out. We have addressed this issue and standardized the point sizes for uniformity.

Q4. In Figure 1e, it is suggested to mark the mutation of A in which positions with red or underline, which is more intuitive.

**Response:** Thank you for your suggestion regarding the visualization of mutations in Figure 1e. We have taken your advice into consideration and have now marked the mutated adenine (A) bases in red to enhance the clarity and intuitiveness of the figure.

**Reviewer #3:** Upon evaluating the revised manuscript and the authors' responses to previous comments, I am pleased to see that the authors have addressed most of the concerns raised in the initial review. The revisions made to the manuscript, particularly in areas of experimental design, data presentation, and addressing specific technical questions, have improved the clarity and scientific rigor of the study. These revisions contribute substantially to the manuscript's quality, making it a valuable addition to the field. Given these improvements and the authors' diligent efforts to address the concerns, I believe that the revised manuscript now meets the publication standards for Genome Biology.

**Response:** we appreciate your positive and constructive comments regarding our manuscript.
